# Characterization and Classification of Cocoa Bean Shells from Different Regions of Venezuela Using HPLC-PDA-MS/MS and Spectrophotometric Techniques Coupled to Chemometric Analysis

**DOI:** 10.3390/foods10081791

**Published:** 2021-08-02

**Authors:** Letricia Barbosa-Pereira, Simona Belviso, Ilario Ferrocino, Olga Rojo-Poveda, Giuseppe Zeppa

**Affiliations:** 1 Department of Agriculture, Forestry and Food Sciences (DISAFA), University of Turin, 10095 Grugliasco, Italy; simona.belviso1@posta.istruzione.it (S.B.); ilario.ferrocino@unito.it (I.F.); Olga.Rojo.Poveda@ulb.be (O.R.-P.); giuseppe.zeppa@unito.it (G.Z.); 2 Department of Analytical Chemistry, Nutrition and Food Science, Faculty of Pharmacy, University of Santiago de Compostela, 15782 Santiago de Compostela, Spain; 3 RD3 Department-Unit of Pharmacognosy, Bioanalysis and Drug Discovery, Faculty of Pharmacy, Université Libre de Bruxelles, 1050 Brussels, Belgium

**Keywords:** cocoa bean shell, fingerprint, polyphenols, methylxanthines, HPLC-PDA-MS/MS, spectrophotometric screening assays, principal component analysis, chemical markers, traceability, antioxidant capacity

## Abstract

The cocoa bean shell (CBS) is one of the main cocoa byproducts with a prospective to be used as a functional food ingredient due to its nutritional and sensory properties. This study aims to define the chemical fingerprint of CBSs obtained from cocoa beans of diverse cultivars and collected in different geographical areas of Venezuela assessed using high-performance liquid chromatography coupled to photodiodes array and mass spectrometry (HPLC-PDA-MS/MS) and spectrophotometric assays combined with multivariate analysis for classification purposes. The study provides a comprehensive fingerprint and quantitative data for 39 compounds, including methylxanthines and several polyphenols, such as flavan-3-ols, procyanidins, and *N*-phenylpropenoyl amino acids. Several key cocoa markers, such as theobromine, epicatechin, quercetin-3-*O*-glucoside, procyanidin_A pentoside_3, and *N*-coumaroyl-l-aspartate_2, were found suitable for the classification of CBS according to their cultivar and origin. Despite the screening methods required a previous purification of the sample, both methodologies appear to be suitable for the classification of CBS with a high correlation between datasets. Finally, preliminary findings on the identification of potential contributors for the radical scavenging activity of CBS were also accomplished to support the valorization of this byproduct as a bioactive ingredient in the production of functional foods.

## 1. Introduction

Several studies have associated the consumption of cocoa and chocolate products with multiple beneficial properties to human health, including inhibition of lipid peroxidation and the protection of LDL-cholesterol against oxidation, reduction of blood pressure, cancer prevention, inflammatory processes modulation in the human body, prevention of the development of metabolic diseases such as obesity and diabetes, etc. [1,2]. These health benefits have been related to the chemical compounds present in these products, mainly polyphenols and alkaloids [3,4]. In addition to their biological activities, theobromine and caffeine; the flavan-3-ols epicatechin and catechin; several procyanidins; and *N*-phenylpropenoyl amino acids have been reported as key non-volatile molecules contributing to the bitter taste and the astringent mouthfeel imparted during consumption of cocoa products [5,6,7]. Additionally, theobromine and caffeine have received considerable attention for their physiological stimulating effects on the central nervous system with beneficial effects on satiety, cognitive function, and mood [2]. Additionally, these molecules have been also related to the sensation of pleasure associated with cocoa consumption and responsible for the enjoyment of the foods/beverages [5].

In recent years, among the several by-products generated during cocoa processing by the industry, cocoa bean shell (CBS) has been one of the main ones considered by researchers to define new strategies of recovery and recycling [8]. Despite the content in proteins, dietary fibers, minerals, and vitamins with nutritional value, CBS also constitutes a potential source of other bioactive compounds, such as polyphenols and methylxanthines [8]. Indeed, several studies have proposed CBS as a low-cost ingredient to develop functional foods, such as bakery [9,10] and dairy products [11] or beverages [12,13]. Furthermore, CBS maintains the aromatic profile characteristic of cocoa that contributes to the pleasant flavor of the final products [14]. Similar to cocoa products, the potential health benefits of these new products developed with CBS, including inhibition of glucose metabolism and antioxidant and anti-inflammatory properties, have been related to the presence of the bioactive compounds, but studies defining the chemical profile of this cocoa by-product and its correlation to the respective bioactivities are limited.

For chemical analysis, UV–vis spectrophotometric assays are the most current methods to estimate the total content of phenolic compounds in foods, including cocoa. [15,16]. Despite the operational simplicity of these screening assays, they also display some limitations, such as the interferences of other substances. Therefore, the employment of more precise and accurate analytical techniques to identify and quantify phenolic compounds in foods is highly recommended [17]. Over the last decade, several authors have defined the chemical profile of cocoa beans and related products mainly based on the polyphenol and methylxanthine fingerprint. For this, several methodologies based on reversed-phase high-performance liquid chromatography (RP-HPLC) coupled to a diode-array detector (DAD) and mass spectrometry (MS) or tandem MS (MS/MS) have been proposed [3,15,18,19,20,21,22,23,24]. Due to the complexity of cocoa extracts and the lack of commercial standards, a sample purification process using solid-phase extraction (SPE) has also been recommended [25,26]. Additionally, the combination of these analytical methodologies with multivariate analysis allows for the classification and discrimination of cocoa and cocoa-related products for the traceability of such products [20,24,27,28,29]. However, the use of these methodologies for the exhaustive characterization of CBS is limited. Indeed, to the best of our knowledge, the recent work performed by Cádiz-Gurrea et al. [30] is the first report on the chemical profile of CBS determined by HPLC-ESI-TOF correlated with that of the respective cocoa beans from Peru.

Venezuela is one of the Caribbean countries producing and exporting the fine or flavored cocoa of *Criollo* and *Trinitario* cultivars, which is highly requested by markets due to its high quality and unique organoleptic properties [31]. Its cocoa production of 25,000 tons per year represents less than 5% of the global production. Since CBS represents 10–17% of the total weight of cocoa beans, every year, up to 4500 tons of CBS can be generated, just from Venezuelan cocoa [32]. Besides the cultivar, other factors such as the geographical origin [29], harvest season, climatic and agronomic conditions, fermentation methods [3,20], and subsequent processing [33] steps (e.g., roasting) [15,34] affect the chemical compositions of cocoa beans and, consequently, final cocoa related products, including CBS. Several chemical compounds might be used as key indicators to certify the quality and to enable the discrimination of cocoa products with a label of origin, with the aim of ensuring food authentication following the new market trend of interest for law enforcement, food producers, importers and exporters, and consumers [35]. Therefore, as for cocoa beans, the study of the CBS chemical fingerprint considering an extended range of compounds is recommended to define the quality and flavor of the product. In addition, it will also contribute to an aware selection of CBS according to the potential health benefits and contribute to its valorization as a functional ingredient. Hence, this work aims to define the chemical fingerprint of CBS by solid-phase extraction combined with high-performance liquid chromatography coupled to tandem mass spectroscopy (SPE-HPLC-PDA-MS/MS) and to determine the compounds responsible for the differences among several CBSs from cocoa beans of different cultivars and geographical areas of Venezuela to allow for the authenticity of this material. Moreover, the applicability of the spectrophotometric assays, such as screening assays, was also evaluated for the classification of the CBS. Finally, the correlation among the datasets obtained was investigated to find the link between the single chemical compounds and their responses to screening assays and their contribution to the antioxidant capacity of the CBS.

## 2. Materials and Methods

### 2.1. Chemicals and Standards

Methanol (≥99.9%), formic acid (98–100%), acetone (≥99.5%), hydrochloric acid (fuming 37%), 6-hydroxy-2,5,7,8-tetramethylchroman-2-carboxylic acid (97%) (Trolox), 2,2-diphenyl-1-picrylhydrazyl (95%) (DPPH), Folin & Ciocalteu’s phenol reagent, sodium carbonate (≥99.5%), vanillin (99%), aluminum chloride (99%), sodium nitrite (≥99%), ethanol (≥99.8%), and sodium hydroxide (1 M) were obtained from Sigma-Aldrich (Milano, Italy). Ultrapure water was prepared in a Milli-Q filter system (Millipore, Milan, Italy). Standards of polyphenols were supplied as follows: (+)-catechin hydrate (>98%), quercetin-3-*O*-glucoside (≥90%), and quercetin (≥98.5%) were provided by Sigma-Aldrich (Milano, Italy); protocatechuic acid (>97%) (-)-epicatechin (>90%), gallic acid (≥98.0%), procyanidin B1 (≥98.5%) (PCB1), procyanidin B2 (≥98.5%) (PCB2), caffeic acid (≥95%), cinnamic acid (≥99.9%), and p-coumaric acid (≥98%) were supplied by Fluka (Milano, Italy). Alkaloids standards (theobromine (≥98.5%) and caffeine (≥98.5%)) were provided by Sigma-Aldrich (Milano, Italy). Stock solutions for all standards were prepared at concentrations of 1 mg/mL in methanol and stored at −20 °C. Working standard solutions were prepared by diluting the stock solutions with HPLC mobile phase (50:50; methanol—water containing 0.1% of formic acid).

### 2.2. Cocoa Bean Shell

Fermented cocoa beans (*Theobroma cacao* L.) from different cocoa-growing areas of Venezuela and cultivars, collected during the seasons of 2014 and 2015, were purchased from several local cocoa companies. In total, 10 samples (2 batches of each) from seven different regions of Venezuela (Sur del Lago, Caucagua, Merida, Cuyagua, Canoabo, Ocumare and Carenero) and two cultivars (*Criollo* (C) (*n* = 5), *Trinitario* (T) (*n* = 5)) were collected (see Figure S1). Specific information related to fermentation and drying conditions is not available since the suppliers retained this information as confidential. Cocoa beans were roasted at 130 °C for 20 min using a ventilated oven Memmert UFE 550 (ENCO, Spinea, Italy). After separation from the beans, CBS samples were ground in an ultra-centrifugal mill Retsch ZM 200 (Retsch Gmbh, Haan, Germany) to obtain a uniform powder with 250 µm of particle size. The humidity content of the CBS (ranging between 5.46–7.44%) was determined using a Gibertini Eurotherm electronic moisture analyzer (Gibertini Elettronica, Novate Milanese MI, Italy). Samples were stored under vacuum at −20 °C before extraction.

### 2.3. Extraction of Bioactive Compounds

For the extraction of polyphenols and methylxanthines extraction, 1.5 g of CBS powder was extracted in 30 mL of an ethanol–water mixture (50:50, *v*/*v*), according to the methodology described by Barbosa-Pereira et al. [36]. Extractions were performed at 25 °C under constant rotatory oscillation (3 oscillations × s^−1^) using a VRL 711 orbital shaker (Asal S.r.l., Milan, Italy) for 2 h. Samples were centrifuged at 4200× *g* using a Heraeus Megafuge 11 Centrifuge (Thermo Fisher Scientific, Hanau, Germany) for 10 min at 4 °C. The extractions were performed in duplicate for all samples, and the supernatants were filtered with a 0.45 μm PTFE filter. Then, 20 mL of each extract was evaporated at 35 °C under constant rotatory agitation at 300 rpm with a flow of nitrogen, using an evaporator/concentrator system Glas-Col^®^ (Glas-Col LLC, Terre Haute, IN, USA). Finally, the dried extract was redissolved in water for the further purification/fractionation step.

### 2.4. Fractionation and Purification of Bioactive Compounds Present in CBS by SPE

SPE fractionation and purification of CBS compounds were performed with Discovery DPA-6S cartridges (Supelco, Bellafonte, PA, USA) containing 500 mg of polyamide sorbent in 6 mL tubes, fitted with frits, in a SPE vacuum manifold of 24 positions (Phenomenex, Castel Maggiore, Italy). Before sample charge, the cartridges were activated with methanol (5 mL) and then preconditioned with the solvent of samples (5 mL of Mili-Q water). Afterward, 500 µL of crude extract prepared in Mili-Q water, as described above in Section 2.3, was loaded on the cartridge. Subsequently, the analytes adsorbed were eluted with different solvent mixtures. Four separated fractions (F) were obtained as follows: 100% water containing 0.1% of formic acid (F1); methanol-water (50:50) (*v*/*v*) (F2); 100% methanol (F3); and finally, 70% acetone-water (70:30) (*v*/*v*) followed by 100% acetone (F4), 5 mL each. The fractionation experiments were performed in duplicate to ensure the repeatability of a final number of 120 fractions (40 for each fraction). All fractions were evaporated to dryness, under nitrogen at 35 °C and constant rotatory agitation at 300 rpm, using an evaporator/concentrator system Glas-Col^®^ (Glas-Col LLC, Terre Haute, IN, USA), and reconstituted with methanol–water containing formic acid 0.1% (50:50) for further analysis (F1 in 1 mL, while F2, F3, and F4 in 200 µL). The fractions yielded were then filtered through a 0.22 μm PTFE filter for HPLC-PDA-ESI-MS/MS analysis. Additionally, the total contents in phenolics, tannins, and flavonoids were assessed by spectrophotometric assays, and the antioxidant capacity of each fraction was measured by the DPPH radical scavenging test.

### 2.5. HPLC-PDA-ESI-MS/MS

Chromatographic analyses were carried out with an HPLC-PDA Thermo-Finnigan Spectra System (Thermo-Finnigan, Waltham, MA, USA). The system was equipped with a P2000 binary gradient pump, SCM 1000 degasser, AS 3000 automatic injector, and Finnigan Surveyor PDA Plus detector.

The CBS compounds were separated on a reverse-phase Kinetex^®^ 5 μm Phenyl-Hexyl column 100 Å, LC column 150 × 4.6 mm (Phenomenex, Castel Maggiore, Italy) equipped with a SecurityGuardTM analytical guard cartridge system (Phenomenex). Ultrapure water containing 0.1% of formic acid (solvent A) and 100% methanol (solvent B) were used as mobile phase. A gradient elution method was applied as follows: 0–2 min, 90% A and 10% B; 2–18 min, linear gradient from 10 to 50% of B; 18–40 min, linear gradient from 50 to 80% B; 40–42 min, linear gradient up to 90% of B; and 42–45 min a linear gradient up to 90% A and 10% of B for column re-equilibration. The column temperature was set at 35 °C. The mobile phase flow rate was 1.0 mL/min. The sample injection volume was 10 μL. PDA spectra were recorded in full scan modality over the wavelength (λ) range of 200 to 400 nm, and PDA chromatograms were extracted at different wavelengths according to the nature of molecules. The instrument control and data collection and processing were performed with ChromQuest software (version 5.0) (Thermo-Finnigan, Waltham, MA, USA).

The MS/MS analysis was performed with an API 3200 QTRAP LC-MS/MS System (Applied Biosystem Sciex, Foster City, CA, USA) equipped with an ESI Turbo V source and a triple quadrupole mass analyzer (Applied Biosystem Sciex) controlled by Analyst software (version 1.6) (AB SCIEX, Redwood City, CA, USA). The detection of chemical compounds was attained in negative ionization mode using the following conditions: ion spray voltage −4500 V; turbo spray temperature 550 °C; curtain gas 2.07 × 10^5^ Pa; interface heater on; nebulizer gas 2.4 × 10^5^ Pa; and heater gas 10 × 10^5^ Pa. Zero air was used as a nebulizer, heater, and eliminator of eluent, while nitrogen was used as curtain and collision gas. The method of full scan mode at high sensitivity, enhanced mass spectrum (EMS), was used for data acquisition, recorded in the range of *m*/*z* 100–1000 amu, operating with the following parameters: declustering potential (DP) of −20 V, entrance potential (EP) of −10 V, and collision energy (CE) of −30 eV. Product ions (MS/MS) were generated according to the information-dependent acquisition (IDA) mode, with a threshold of 50,000 cps and a collision energy (CE) of −30 eV, collected in an enhanced product ion (EPI) mode. For methylxanthines, mass spectrometry analyses were carried out under the same conditions described above for the other chemical compounds, but in this case, the detection was accomplished in positive ionization mode.

### 2.6. Identification and Quantification of Chemical Compounds in CBS

Tentative identification of chemical compounds was accomplished by comparing their UV–vis spectrum, molecular ion [M-H]^−/+^, and mass spectrometry fragmentation pattern (MS/MS) with those already described in the literature. When commercial standards were available, the individual phenolic compounds were identified by comparing the retention times and UV–vis spectra (λ_max_) with those obtained by injecting pure standards under the same HPLC conditions and with molecular ion and MS fragmentation data provided by MS/MS analysis. Standard solutions of each compound were prepared in a 50:50 (*v*/*v*) methanol–water containing 0.1% of formic acid mixture. The solutions were injected individually into the HPLC column and eluted under the same analytical conditions described above to determine their chromatographic retention times and to collect UV spectra by the DAD detector and MS fragmentation data. The quantification of each phenolic compound and methylxanthines was made by the external standard method using seven-point regression curves constructed at the wavelength of maximum absorbance of each analyte. Limits of detection (LOD) and quantification (LOQ) for the polyphenols and methylxanthines were calculated by multiplying the signal of the noise of the blanks by 3 and 10 times, respectively. For those polyphenols for which commercial standards were not available, quantification was performed as equivalents of phenolic compounds related to the corresponding family group of molecules. The content of polyphenols and methylxanthines present in CBSs and their fractions was expressed as mg/kg dry weight (dw) and g/kg dw of CBS, respectively.

### 2.7. Total Phenolics, Total Flavonoids, and Total Tannins

The total phenolic content (TPC), total flavonoid content (TFC), and total tannin content (TTC) were assessed according to the spectrometric assays described by Barbosa-Pereira et al. [36]. All the determinations were performed in triplicate using 96-well microplates. The corresponding absorbance was recorded using a BioTek Synergy HT spectrophotometric multi-detection microplate reader (BioTek Instruments, Milan, Italy). Quantification of TPC was carried out using a standard curve of commercial gallic acid (20–100 mg/L, R^2^ = 0.9981), and the concentration was expressed as mg of gallic acid equivalents (GAE)/g of CBS, while for TFC and TTC, the results were calculated based on the standard curve of catechin (5–500 mg/L, R^2^ = 0.9980) and expressed as mg of catechin equivalents (CE)/kg of CBS.

### 2.8. Antioxidant Capacity

The antioxidant capacity of extracted polyphenols present in CBSs and its fractions was determined by the 2,2′-diphenyl-1-picrylhydrazyl (DPPH·) radical scavenging assay described by Barbosa-Pereira et al. [36]. All determinations were performed in triplicate using 96-well microplates, and the absorbance was measured at 515 nm using a BioTek Synergy HT spectrophotometric multi-detection microplate reader (BioTek Instruments, Milan, Italy). The inhibition percentage (IP) of the radical DPPH was calculated using the following equation:

IP (%) = (A_0_ − A_30_)/A_0_ × 100, (1)

where A_0_ is the absorbance at the initial time, and A_30_ is the absorbance after 30 min. A linear regression curve of Trolox was used at 12.5–300 μM (R^2^ = 0.9988) to calculate the radical scavenging activity (RSA) values. Results were expressed as mmol of Trolox equivalents (TE)/kg of CBS.

### 2.9. Chemometrics and Statistical Analysis

To discriminate the Venezuela CBS samples as a function of the geographic area of origin and cultivar, principal-component analysis (PCA) plots based on the normalized data (log_10_) were built by using the *made4* package of R (https://www.r-project.org) and the function dudi.pca. Analysis of similarity based on phenolic molecules and spectrophotometric assays was applied with 999 permutations to detect significant differences as a function of geographical origin or cultivar, by using the anosim function in *vegan* package of R. Non-parametric Kruskal–Wallis and Wilcoxon tests were carried out to find chemical compounds differentially abundant between all the variables. Data were visualized as box plots representing the interquartile range between the first and the third quartile, with the error bars showing the lowest and the highest value. Pairwise Spearman’s non-parametric correlations (corr.test function in *psych* package of R) were used to study the relationships between bioactive compounds and spectrophotometric assays and antioxidant capacity. The correlation plots were visualized in R using the *corrplot* package of R. *p*-values were adjusted for multiple testing, and a false discovery rate (FDR) <0.05 or lower was considered as statistically significant.

## 3. Results and Discussion

A comprehensive characterization of chemical compounds of CBSs from Venezuela was performed to define the chemical fingerprint for the valorization of this cocoa byproduct as a food ingredient due to its bioactivities and sensorial properties. Thus, the present study was divided into two main sections: (1) one dedicated to the chemical characterization by advanced HPLC-PDA-MS/MS and rapid screening spectrophotometric assays, (2) followed by the chemometric analysis and the identification of key markers, which allowed the classification and authentication of CBS.

### 3.1. Chemical Profile of CBS Characterized by HPLC-PDA-MS/MS

The chemical compounds of CBS samples from different growing regions of Venezuela, identified or tentatively identified by HPLC-PDA-MS/MS, are described in Table 1. The fractionation of the raw extract by SPE allowed different fractions to be yielded and contributed to an easier chromatographic separation, identification, and classification of the bioactive compounds. The chromatograms of a representative CBS raw extract and their fractions obtained by HPLC-PDA-MS/MS are shown in Figure S2. The compounds were characterized by their retention times (R_t_), the UV–vis spectrum, and the characteristic maximum wavelength, molecular formula, molecular ion [M-H], and mass spectrometry fragmentation pattern (MS/MS) together with those already described in the literature. The confirmation of the identity of some compounds was achieved by comparing with data obtained by injecting pure standards under the same HPLC-PDA-MS/MS conditions (see Table 1). Since studies on the chemical profile of cocoa by-products such as CBS by HPLC-MS/MS are limited or inexistent in the literature, the tentative identification of the chemical compounds will be discussed by comparing data with studies performed in cocoa beans and cocoa products and with the recent study reported by Cádiz-Gurrea et al. [30] on CBS from cocoa beans with origin in Peru. 

#### 3.1.1. Methylxanthines

The alkaloids, theobromine and caffeine (compound **2** and **19**, respectively), detected at UV λ_max_ of 272 nm, were the most representative group of chemical compounds identified in the CBS samples. The identification was achieved by comparing the retention times and MS/MS fragmentation, acquired in positive mode with commercial standards. These methylxanthines were isolated in the first fraction (F1) yielded from the SPE fractionation process of CBS raw extract with water (see Figure S2). Theobromine, with [M-H]^+^ ion at *m*/*z* 181, and MS/MS fragments 167, 138, and 137 *m*/*z*, and caffeine, with [M-H]^+^ ion at *m*/*z* 195, and MS/MS fragments 181 and 138 *m*/*z*, are the main representative alkaloids found in cocoa and cocoa-related products, including cocoa bean shell [3,18,21,22,37].

#### 3.1.2. Phenolic Acids

The compound **1** (λ_max_ of 293 nm, R_t_ 4.73 min), with [M-H]^−^ ion at *m*/*z* 153, MS/MS fragment 109 *m*/*z*, was identified as protocatechuic acid. The retention time and MS/MS fragmentation pattern obtained with the reference standard confirmed the identity of the phenolic acid. Protocatechuic acid is a hydroxybenzoic acid derivate, widely distributed in nature, reported for cocoa beans and related products in several studies [22,23]. Recently, this compound was also described in cocoa bean husks from Peru [30], Colombia [12], Honduras [38], and Ecuador [11,13].

#### 3.1.3. Flavan-3-ols and Their Glycosides

Compounds **7** and **16**, with characteristic UV–visible spectra and λ_max_ at 278 nm, [M-H]^−^ ion at *m*/*z* 289, and MS/MS fragments 245, 205, and 203 *m*/*z*, were identified in the CBS as catechin and epicatechin, respectively, and their identity was confirmed by comparison with pure standards. These compounds have been recognized as the main compounds of the cocoa bean, cocoa products, and cocoa byproducts [3,20,23,36].

Compound **4** and compound **9**, with [M-H]^−^ ion at *m*/*z* 451 and the MS/MS fragments 289 and 245 *m*/*z* from catechin, acquired at different retention times, 7.3 min and 9.5 min, were tentatively identified as two isomers of flavan-3-ols glycosides, catechin-3-O-glucoside_1 and catechin-3-O-glucoside_2, respectively. These catechin glycosides were described for cocoa beans in studies performed by Patras et al. [19] and Cádiz-Gurrea et al. [30]. Both compounds were identified in CBS samples by HPLC-PDA-MS/MS for the first time in this study.

#### 3.1.4. Procyanidins

In addition to the monomers of flavan-3-ols and their glycosides, several oligomers (procyanidins) and their derivates composed of several unities of catechin and epicatechin were also identified in the CBS samples. A total of 18 compounds, such as B-type procyanidin dimers and trimers and A-type procyanidin glycosides, were tentatively identified among the several fractions yielded from the CBS raw extracts.

Four chemical compounds (compounds **14**, **15**, **22**, and **26**) with the same UV–visible spectra λ_max_ at 280, [M-H]^−^ ion at *m*/*z* 577, and the fragmentation pattern that results in the fragment 289 *m*/*z*, equivalent to the monomers of catechin/epicatechin, were identified as B-type procyanidin dimers. Some of these compounds have been described in the literature in cocoa beans and other cocoa products such as cocoa husks and beans from Peru [19,30]. In addition, eight chemical compounds (**11**, **13**, **21**, **24**, **25**, **27**, **29**, and **31**), with λ_max_ at 278–280 nm, [M-H]^−^ ion at *m*/*z* 865, and the same fragmentation pattern in negative ionization mode that results in the fragments 577, 425, 407, and 289 *m*/*z*, were identified as B-type procyanidins trimers [39]. As for procyanidins B-type dimers, the same studies mentioned above described these types of polyphenols for cocoa beans and cocoa products. This study describes, for the first time, the identification of B-type procyanidin trimers in CBS. Finally, six compounds (**23**, **28**, **30**, **32**, **33**, and **35**), with the UV–visible spectra typical at λ_max_ 280 nm were identified as A-type procyanidins glycosides. Compounds **23**, **30,** and **35**, with [M-H]^−^ ion at *m*/*z* 707 and MS/MS fragments 581, 539, 449, and 289 *m*/*z*, were identified as isomers of Procyanidin A-type pentosides (1, 2, and 3, respectively) [19]. On the other hand, compound **28** and compound **33**, with [M-H]^−^ ion at *m*/*z* 737 and a similar fragmentation pattern that results in the fragments 611, 539, 449, and 289 *m*/*z*, were identified as isomers of procyanidin A-type hexoside [20,40]. Additionally, compound **32**, with [M-H]^−^ ion at *m*/*z* 995, which displayed the fragmentation pattern in negative ionization mode that results in the fragments 865 and 407 *m*/*z*, was identified as procyanidin A-type trimer arabinoside, as described by D’Souza et al. [20] for fermented cocoa beans. These compounds were not described previously in the literature for cocoa bean shells.

#### 3.1.5. Flavonols and Their Glycosides

The UV–visible spectra typical at λ_max_ 255 nm and 355 nm and the similar fragmentation pattern in negative ionization mode that results in the fragment 301 *m*/*z* determined that compounds **36** and **38** were the flavonol glycosides quercetin-3-*O*-glucoside ([M-H]^−^ ion at *m*/*z* 463) and quercetin-3-*O*-arabinoside ([M-H]^−^ ion at *m*/*z* 433), respectively. The standard of quercetin-3-*O*-glucoside (compound **36**) allowed confirm the identity of these flavonols to be confirmed. The identification of the aglycone quercetin (compound **39**) was achieved by comparing the data with those obtained from the authentic standard, at λ_max_ 275 and 365 nm. On the other hand, compound **37**, with a [M-H]^−^ ion at *m*/*z* 593, was tentatively identified as kaempferol-3-*O*-rutinoside, based on the MS/MS fragment 285 *m*/*z* of kaempferol, based on the UV–visible spectra typical at λ_max_ 266 and 345 nm, and supported by the literature where this flavonol glycoside was described before for cocoa beans [20,22].

#### 3.1.6. N-Phenylpropenoyl-L-Amino Acids

Compounds **3** and **12**, with [M-H]^−^ ion at *m*/*z* 278, MS/MS fragments 234, 163, and 119 *m*/*z* and the UV–visible spectra typical at λ_max_ 279/284 and 309/306 nm, were identified as *N*-Coumaroyl-L-aspartate isomers 1 and 2, respectively. Together with compounds **17** and **34**, these molecules displayed a similar fragmentation pattern in negative ionization mode that is characteristic of the coumaric acid (fragments 163, 132, 119 *m*/*z*) [37]. The tentative identification of *N*-coumaroyl-L-glutamate (compound **17**) was achieved based on the [M-H]^−^ ion at *m*/*z* 292 and the MS/MS fragmentation pattern 274, 248, 230, 202, 163, 143, and 119 *m*/*z*, as described for cocoa beans, and the UV–visible spectra typical at λ_max_ 286 nm [7]. *N*-coumaroyl-L-tyrosine (Deoxyclovamide) (compound **34**) was tentatively identified based on the [M-H]^−^ ion at *m*/*z* 326; the characteristic MS/MS fragments 282, 206, 163, 145, 134, and 119 *m*/*z*; and its UV–visible spectra typical at λ_max_ 283 nm, as described by several authors in the literature for cocoa products [37].

Besides the coumaric acid derivates, *N*-caffeoyl-L-aspartate was also tentatively identified (compound **6**) based on the [M-H]^−^ ion at *m*/*z* 294; MS/MS fragments 276, 179, and 132 *m*/*z*; and the UV–visible spectra typical at λ_max_ 282 and 320 nm. Part of the identity was confirmed based on the fragmentation patterns obtained with the pure standard of caffeic acid, 179 and 132 *m*/*z*. Finally, compound **20**, with [M-H]^−^ ion at m/z 308; MS/MS 276, 264, 246, 193, 149, and 134 *m*/*z*; and the UV–visible spectra typical at λ_max_ 286 and 318 nm, was tentatively identified as *N*-Feruloyl-L-aspartate due to the fragmentation pattern characteristic of ferulic acid, 193 and 134 *m*/*z*. The hydroxycinnamic acids coumaric, caffeic, and ferulic acids’ MS/MS fragmentation patterns were confirmed by comparison with the data obtained from the analysis of pure reference standards. *N*-Coumaroyl-l-glutamate and *N*-Feruloyl-l-aspartate were described before for cocoa beans [7] but are described for the first time for CBS in the present work.

#### 3.1.7. Others

Among the compounds described and classified above, two isomers of sulfated compounds containing sulfonic acid [19], compounds **5** (C_11_H_21_O_9_S_1) and **8** (C_11_H_21_O_9_S_2), with UV–visible spectra typical at λ_max_ 278 nm, were tentatively identified based on the [M-H]^−^ ion at *m*/*z* 329 and MS/MS fragments 241. Additionally, compound **18** (C_12_H_18_O_7_S), with the UV–visible spectra λ_max_ 275 nm, was tentatively identified as hydroxyjasmonic acid sulfate, due to the [M-H]^−^ ion at *m*/*z* 305 and MS/MS fragments 225, as described before in the literature for fermented and unfermented cocoa beans [19,20]. Compound **10** with λ_max_ 293 nm and [M-H]^−^ ion at *m*/*z* 357 was tentatively identified as Sweroside, as described by others in the literature for cocoa products [26].

### 3.2. Quantitative Distribution of Bioactive Compounds in CBS and Antioxidant Capacity

The quantification of 11 chemical compounds was performed based on the calibration curves prepared with external standards, according to the analytical parameters described in Table S1. The other compounds were tentatively quantified according to the similarity of the molecular structure as follows: procyanidins expressed as equivalents of procyanidin B1; catechin-3-*O*-glycosides as equivalents of catechin; flavonol-3-*O*-glycosides as equivalents of quercetin-3-*O*-glucoside; and *N*-phenylpropanoid-l-amino acids as equivalents of the respective phenolic acid present in the structure, *p*-coumaric, caffeic acid, and ferulic acids. The amounts of each molecule and total amounts of each group of molecules quantified for the several CBS samples are in Table 2. The amounts determined for each CBS quantified in the single fractions are in Tables S2, S3, S4, and S5 for fractions F1, F2, F3, and F4, respectively.

Among the 39 chemical compounds identified in CBS samples, methylxanthines were the most representative group found in the range of 5.5 and 15.7 g/kg of CBS. As described for cocoa products, theobromine was present in higher concentrations (4.66–9.95 g/kg) than caffeine (0.84–5.80 g/kg) [33]. Furthermore, the CBS samples of *Criollo* cultivar were those with high amounts of both compounds, and these results are in agreement with those described in the literature for cocoa beans and related products, including CBS [36,41]. Considering the ratios of theobromine/caffeine proposed by several authors for the classification of cocoa cultivars, in this study, this ratio was considered for the first time for CBS, and the results showed similar values between 1.71 and 5.54 g/kg [21]. These values were 10-fold higher than those described in the literature for cocoa beans [22,33] and lower than that reported by others [21,27]. However, the results are in line with those reported for cocoa bean shells in recent years [36,42]. CBSs from the *Criollo* cultivar showed lower theobromine/caffeine ratios than those observed for CBSs from the *Trinitario* cultivar. The average value to discriminate CBS *Criollo* from *Trinitario* cultivars was around 2, since the values for *Criollo* cultivars ranged between 1.7 and 1.9, while for the *Trinitario* cultivar, the ratio ranged between 1.9 and 5.5. This mean value of ratio was lower than that described in the literature for cocoa beans, where values above 3 are characteristic for *Trinitario* cocoa beans, and values below 3 are characteristic of the *Criollo* cultivar [21]. Another observation from the CBS analysis was that the percentage of caffeine within the range 0.08–0.5% was higher in samples of CBS from the *Criollo* cultivar (0.4–0.5%). These contents were similar to those described in the literature by Pedan et al. [21] and higher than those described by Carrillo et al. [27]. The methylxanthines have been related positively to the acceptance of cocoa products by consumers because of their effect on the central nervous system due to their stimulating properties and their mood improvements, among other positive effects on the cardiovascular system or in preventing intestinal inflammation [43]. Thus, CBS constitutes an important source of these bioactive compounds, the CBSs from the *Criollo* cultivar being those with high interest. These compounds might be isolated with the SPE purification process proposed in this study in fraction F1 (Table S2).

The other group of chemical compounds (*n* = 37), quantified in this study by HPLC-PDA, was composed mainly of phenolic compounds in the range between 237.1 and 1319.6 mg/kg of CBS. CBSs yielded from the *Trinitario* cultivar cocoa beans produced in the regions of Merida and Cayagua were those with high amounts of these bioactive compounds, 1319.6 and 1134.9 mg/kg, respectively. On the other hand, CBSs of the *Criollo* cultivar from the Carenero and Ocumare regions were those with low contents in phenolic compounds (367.4 and 237.1 mg/kg, respectively). The sum of the fractions F2, F3, and F4 (see Tables S3–S5) contributed with 16.9%, 35.5%, and 46.7%, respectively, to the total amount.

Phenolic acids contribute with 8.3% (average value) to the total amount of phenolics in CBS analyzed in this study. Notwithstanding the recent literature reporting other phenolic acids in CBS from Peru [30], protocatechuic acid was the only compound identified in CBSs from Venezuela. In general, the CBSs samples yielded from cocoa beans of the *Trinitario* cultivar were those with high contents in protocatechuic acid (53.95–113.8 mg/kg). The contents of protocatechuic acid found in this study for the CBS were higher than those found by Rodríguez-Carrasco et al. [22] for the respective cocoa beans from these three origins and cultivars from Venezuela, Canoabo_C, Ocumare_C, and Sur del Lago_T (17.3, 23.7, and 28.8 mg/kg, respectively).

The most representative group of polyphenols present in cocoa products, and therefore also in CBS yielded from cocoa beans from different regions of Venezuela studied in this work, was flavonoids (around 63–82%, average 71.57%), comprising flavan-3-ols and their derivates, procyanidins, and flavanols and their respective glycosides. These values were similar to those described in the literature for cocoa beans and their products [22,23].

The flavan-3-ols catechin and epicatechin were found to be the main compounds in CBS samples, representing around 23% of the total content of polyphenols in *Trinitario* CBS and 10% for the *Criollo* cultivar. These compounds were purified by SPE and isolated in fraction 3 (Table S4). Epicatechin was found in higher concentrations in all samples, the samples Merida_1_T and Cayagua_T CBSs being those with high amounts reaching 298.0 and 234.2 mg/kg, respectively. These values are in agreement with the lowest values described in the literature for CBS from different geographical origins [33,36] and were lower than those observed by other studies on cocoa beans [22]. The ratio of epicatechin/catechin (E/C) was also determined for all CBS samples, for the first time in this study, in the range from 5.0 for Cayagua_T samples to 10.0 in Sur del Lago_T. This ratio was previously described by Ioannone et al. [15] for cocoa beans during the roasting process (E/C < 1) and by Damm et al. [18] for chocolate and cocoa samples, in the range from 2.2 to 4.0. These authors also observed that the ratio was not constant among samples and is dependent on the processing conditions. Indeed, considering the data described by Rodríguez-Carrasco et al. [22], the epicatechin/catechin ratios observed for the cocoa beans analyzed from the same geographic areas of Venezuela used in this study, Canoabo_C, Ocumare_C, and Sur del Lago_T, were 1.5, 1.6, and 1.7, respectively, while for the respective CBS analyzed in this study, the values were 6.3, 6.2, and 10.0, respectively. Thus, since the results obtained from CBS samples were significantly higher than cocoa beans, this ratio might be considered a parameter to discriminate CBS from cocoa beans and other cocoa products.

Derivates of flavan-3-ols, the glycosides of catechin, catechin-3-*O*-glucoside_1, and catechin-3-*O*-glucoside_2, were also quantified. The sum of these isomeric compounds represents 6% of the total polyphenols present in the CBS that were randomly distributed among all CBS samples analyzed in the range from 17.70 mg/kg of CBS (Canoabo_C) to 96.98 mg/kg of CBS (Cuyagua_T). These compounds were purified by SPE, isolated in fraction F2 (Table S3), and identified and quantified for the first time for CBS of Venezuela.

The total content in oligomers of flavan-3-ols, classified in procyanidins B-type (PCB), PCB trimers, and procyanidins A-type (PCA) glycosides, represents between 32% and 54% of the total amounts of phenolic compounds present in CBS. Among the three groups of PCBs, the PCB trimers were found in higher amounts from 77.66 mg/kg of CBS (Ocumare_2_C) up to 346.5 mg/kg (Merida_1_T) and represent 22.5% of the total content of polyphenols in CBS, while PCBs and PCA glycosides represent around 7.3 and 13.2% of the total amount of chemical compounds, respectively. Regarding the PCBs, PCB_1 was the isomer found in higher amounts, within the range from 6.29 to 48.47 mg/kg, and the CBS samples yielded from cocoa beans from the *Trinitario* cultivar were those with high concentrations. Considering the PCA glycosides, CBS samples obtained from cocoa beans from the geographical area of Merida were those with high amounts of these compounds (124.4–150.0 mg/kg), in particular PCA pentoside_3 (39.06–71.61 mg/kg). Despite these three groups of PCBs being randomly distributed, considering their sum, the total amount of PCBs was found to be higher for almost all CBSs of the *Trinitario* cultivar if compared with the *Criollo* cultivar. The total amount of PCBs in CBS samples of Canoabo_C and Ocumare_2_C was higher than that described for the cocoa beans of the same geographical area by Rodríguez-Carrasco et al. [22], while for CBS samples from Sur del Lago_T, the values were lower for CBS than cocoa beans. However, in this study, a high number of compounds were quantified, compared to the work performed for the cocoa beans, and other compounds might contribute differently to the final amounts.

Flavonols (quercetin) and their glycosides were also quantified for CBS in the range from 5.64 mg/kg of CBS (Ocumare_2_C) to 61.5 mg/kg (Ocumare_1_T), but their contribution for the total amounts of phenolic compounds was around 3.8%, and the contribution of quercetin alone represents less than 0.5% for all CBS samples from Venezuela. The CBSs with high contents of flavonols were those yielded from cocoa beans of the *Trinitario* cultivar, the quercetin-3-*O*-arabinoside being the most relevant, as described before in the literature for cocoa beans [34]. The total contents determined in CBS samples were similar or higher than that reported by Rodríguez-Carrasco et al. [22] for the respective cocoa beans and those described by Damm et al. [18] for other cocoa products, despite the heterogenicity of the individual compounds among the studies.

Another group of chemical compounds found in the CBS samples was the *N*-phenylpropenoyl-L-amino acids that have been proposed as cocoa product chemical markers. These compounds represent 12.6% (from 4.1% to 19.5%) of the total amount of bioactive compounds, in the range from 26.6 mg/kg of CBS (Ocumare_2_C) to 217.9 mg/kg of CBS (Ocumare_1_T). Among them, the pattern *N*-caffeoyl-L-aspartate > *N*-coumaroyl-L-aspartate_2 > *N*-coumaroyl-L-tyrosine was followed for all CBS samples, similar to that described by others for cocoa beans and related products [3,18].

Finally, a small group of chemicals that contributes 7.5% to the total content of bioactive compounds was also quantified for the first time for CBS in the range from 11.93 mg/kg (Ocumare_2_C) to 114.4 mg/kg (Ocumare_1_T). The compounds C_11_H_21_O_9_S_2 and hydroxyjasmonic acid sulfate were those found in high amounts among all CBS samples. 

From the data obtained and compared with the study performed on cocoa beans, it was noticed that the chemical profile of CBS is similar to that of cocoa beans. However, the proportions of some families of compounds change substantially among them. Cocoa beans display higher contents of flavan-3-ols (47–53%) than PCBs (26–35%) [22], while for CBS samples, the amount in PCBs is higher (36–54%) than flavan-3-ols (9–25%). Additionally, the phenolic acids showed a higher contribution for the total compositions of bioactive compounds of CBS (4–14%) than for cocoa beans (2–9%). These data allow the discrimination of CBSs from their related cocoa bean and might be of great interest for the industry for authentication purposes and for avoiding falsifications in cocoa powder.

Parallel to the HPLC-PDA-MS/MS analysis, spectrophotometric assays, including total phenolic content (TPC), total flavonoid content (TFC), and total tannins content (TTC), were also performed to characterize the CBS samples from Venezuela. The radical scavenging activity (RSA) of the CBS extracts was also assessed with the DPPH assay. All fractions yielded from the SPE purification were evaluated separately for each spectrophotometric assay. TPC values of CBS ranged from 5.87 g GAE/kg of CBS (Carenero_C) to 9.12 g GAE/kg of CBS (Caucagua_T), which was 10-fold higher than the total amount of phenolics determined by chromatography. These results were in line with previous studies performed for CBS and cocoa beans from different origins, including Venezuela (5–12 g GAE/kg) [27,36]. The contributions of each fraction for the total value of TPC were on average of 43.1%, 17.6%, 14.1%, and 25.1% for F1, F2, F3, and F4, respectively. However, the main compounds identified and quantified in fraction F1 were the methylxanthines, and phenolic compounds were not identified. These results support the concerns recently highlighted by Granato et al. [17] related to the interferences of sample components that lead to overestimation values in some screening methods. On the other hand, the results obtained with the TFC and TTC assays were more accurate. In the case of TFC, the values ranged from 1.89 g CE/kg of CBS (Caranero_C) to 3.65 g CE/kg of CBS (Merida_1_T) against 0.29 and 1.01 g/kg of CBS determined by chromatographic analysis for the same samples, respectively. The contributions of the fractions for the total TFC value were 11.7, 23.5, 24.4%, and 41.7% for F1, F2, F3, and F4, respectively. The last three fractions contain the flavonoids determined by chromatography (Tables S3–S5). Similar results were obtained with the TTC method (TTC values ranged from 1.05 to 1.75 g CE/kg of CBS), where the average contributions were around 19.06%, 10.48%, 33.82%, and 34.99% for F1, F2, F3, and F4, respectively. Fractions F3 and F4 were those with high amounts of procyanidins (Tables S4 and S5) quantified by HPLC-PDA (up to 0.56 g/kg of CBS), but considering all flavan-3-ols, the final content of 0.93 g/kg of CBS was reached.

Regarding the antioxidant capacity (RSA) of the CBS, the results showed RSA values in the range of 17.33 mmol TE/kg of CBS (Carenero_C) and 26.33 mmol TE/kg of CBS (Caucagua_T). Additionally, with this methodology, the antioxidant capacity could be overestimated considering the results obtained in all fractions. According to the data, fraction F1 contributes to 28.6% of the total antioxidant capacity, despite the chromatographic analysis not being able to identify the bioactive compounds. Considering the contribution of fractions F2, F3, and F4 to the total RSA of CBS samples, 18.6%, 16.9%, and 34.7%, respectively, the results might allow for a high correlation between the RSA and the bioactive compounds present in each fraction. These methods showed a moderate correlation among them (RSA/TPC, *r* = 0.83; RSA/TFC, *r* = 0.69; RSA/TTC, *r* = 0.69). However, the results were lower than those observed previously by the authors for CBS raw extracts without purification processes [13,36].

**Table 2:** Total and individual contents of chemical compounds and spectrophotometric assay results of CBS samples (*n* = 10) yielded from cocoa beans from seven different regions of Venezuela and two cultivars, *Trinitario* (*n* = 5) and *Criollo* (*n* = 5). Each origin contains 4 samples from 2 different batches (*n* = 40). The content of bioactive compounds for each sample results from the sum of the quantification of the four fractions yielded individually (*n* = 120). Data are expressed as mg/kg dw of CBS, except for methylxathines, which are expressed as g/kg dw of CBS.

Compound	Sur del Lago_T			Caucagua_T			Merida_1_T			Cuyagua_T			Ocumare_1_T			Canoabo_C			Merida_2_C			Merida_3_C			Carenero_C			Ocumare_2_C		
**Phenolic acids**																														
Protocatechuic acid	69.63	±	9.38	53.95	±	10.31	103.1	±	14.44	113.8	±	15.94	84.13	±	17.41	111.4	±	21.98	43.65	±	9.61	46.88	±	7.39	29.91	±	4.16	12.07	±	0.36
**Σ**	**69.63**			**53.95**			**103.1**			**113.8**			**84.13**			**111.4**			**43.65**			**46.88**			**29.91**			**12.07**		
**Flavan-3-ols**																														
Catechin	17.04	±	4.72	16.27	±	3.42	48.31	±	4.76	46.87	±	9.88	32.43	±	1.40	22.05	±	5.04	10.73	±	2.37	11.08	±	2.41	6.04	±	1.37	5.04	±	1.09
Epicatechin	170.9	±	40.2	102.3	±	29.04	298.0	±	22.53	234.2	±	7.87	200.8	±	13.59	140.7	±	32.69	65.61	±	15.6	61.00	±	13.5	30.59	±	7.11	31.23	±	7.40
**Σ**	**187.9**			**118.5**			**346.3**			**281.1**			**233.2**			**162.7**			**76.34**			**72.07**			**36.64**			**36.27**		
**Catechin-3-O-glycosides**																														
Catechin-3-O-glucoside_1	77.67	±	23.5	84.64	±	33.01	22.70	±	1.23	90.02	±	15.74	32.17	±	3.92	17.70	±	7.70	17.85	±	5.33	17.68	±	8.45	22.51	±	7.67	33.79	±	5.13
Catechin-3-O-glucoside_2	1.95	±	0.67	1.75	±	0.77	1.49	±	0.09	6.96	±	2.98	8.05	±	3.51	n.d.			5.61	±	1.73	2.44	±	0.14	1.22	±	0.50	0.90	±	0.16
**Σ**	**79.62**			**86.39**			**24.19**			**96.98**			**40.22**			**17.70**			**23.46**			**20.12**			**23.73**			**34.69**		
**Procyanidins B-type (PCB)**																														
PCB_1	36.63	±	11.2	20.88	±	5.33	48.47	±	1.28	45.91	±	8.97	42.15	±	2.65	37.78	±	15.82	34.94	±	13.5	25.81	±	5.72	16.29	±	7.90	6.29	±	1.63
PCB_2	12.57	±	5.62	21.27	±	3.28	25.56	±	2.47	25.23	±	1.63	17.02	±	3.96	11.92	±	4.41	42.36	±	3.63	11.69	±	0.84	8.73	±	2.39	1.33	±	0.18
PCB_3	5.06	±	1.05	2.29	±	0.58	4.92	±	0.25	6.11	±	0.70	3.42	±	0.46	3.72	±	1.30	4.47	±	0.49	2.37	±	0.49	2.14	±	0.75	0.56	±	0.15
PCB_4	5.03	±	0.38	5.48	±	2.24	5.40	±	2.04	8.54	±	5.53	10.81	±	2.70	3.97	±	0.92	2.61	±	0.99	1.13	±	0.51	0.85	±	0.17	1.18	±	0.20
**Σ**	**59.30**			**49.91**			**84.36**			**85.78**			**73.40**			**57.38**			**84.37**			**41.00**			**28.02**			**9.36**		
**Procyanidins B-type (PCB) trimers**																														
PCB trimer_1	4.68	±	1.58	7.22	±	1.89	9.32	±	1.37	14.27	±	1.15	13.95	±	0.76	6.06	±	0.79	8.44	±	2.62	7.05	±	2.75	4.36	±	1.77	3.02	±	1.35
PCB trimer_2	78.93	±	21.9	45.92	±	12.72	130.5	±	15.03	99.60	±	6.92	79.64	±	4.88	59.52	±	11.26	49.22	±	7.87	40.67	±	10.3	38.43	±	13.4	46.11	±	8.21
PCB trimer_3	4.19	±	3.12	n.d.			n.d.			n.d.			n.d.			2.18	±	0.67	16.64	±	8.75	22.65	±	8.20	13.98	±	5.02	5.43	±	3.62
PCB trimer_4	58.78	±	14.0	21.07	±	6.01	78.01	±	47.46	47.46	±	6.41	38.17	±	2.68	39.53	±	8.58	2.96	±	4.28	3.30	±	3.14	0.74	±	0.79	0.40	±	0.44
PCB trimer_5	8.09	±	2.34	10.46	±	2.38	3.67	±	0.54	2.95	±	0.98	4.81	±	0.63	4.75	±	1.25	7.60	±	3.90	1.85	±	0.77	1.27	±	0.22	1.28	±	0.24
PCB trimer_6	49.33	±	9.95	18.16	±	5.37	51.95	±	11.43	30.70	±	5.70	22.95	±	2.43	28.59	±	6.41	32.50	±	9.52	33.03	±	12.5	17.95	±	2.82	15.81	±	3.08
PCB trimer_7	8.10	±	1.68	n.d.			41.51	±	5.18	18.78	±	4.43	22.13	±	1.08	6.42	±	1.41	2.89	±	0.22	1.37	±	0.38	1.37	±	0.56	0.99	±	0.40
PCB trimer_8	21.49	±	3.52	11.91	±	3.86	31.49	±	3.99	20.02	±	3.55	22.33	±	4.01	17.89	±	1.72	12.66	±	5.26	13.60	±	3.70	10.40	±	2.54	4.62	±	0.56
**Σ**	**233.6**			**114.7**			**346.5**			**233.8**			**204.0**			**165.0**			**132.9**			**123.5**			**88.49**			**77.66**		
**Procyanidins A-type (PCA) glycosides**																														
PCA pentoside_1	1.32	±	0.51	2.32	±	0.50	1.89	±	0.13	2.00	±	0.38	1.94	±	0.48	1.69	±	0.15	0.88	±	0.12	0.84	±	0.45	0.42	±	0.02	0.59	±	0.18
PCA pentoside_2	13.61	±	2.16	16.06	±	4.03	27.66	±	4.66	19.58	±	6.14	24.55	±	3.32	14.45	±	2.73	19.88	±	5.29	28.54	±	13.0	16.74	±	5.36	8.64	±	2.39
PCA pentoside_3	14.74	±	1.39	8.43	±	3.46	39.06	±	2.48	18.95	±	3.48	17.96	±	1.49	22.79	±	2.62	52.78	±	13.7	71.61	±	14.6	47.32	±	7.71	15.03	±	3.71
PCA hexoside_1	n.d.			n.d.			n.d.			n.d.			n.d.			n.d.			22.42	±	4.00	14.46	±	3.66	11.43	±	2.16	1.03	±	0.05
PCA hexoside_2	18.86	±	2.24	11.71	±	5.30	37.11	±	3.75	21.35	±	3.91	24.76	±	2.37	22.98	±	3.67	13.15	±	2.84	9.61	±	1.77	13.32	±	1.35	3.76	±	0.61
PCA trimer arabinoside	24.03	±	1.65	12.85	±	3.40	24.56	±	2.53	17.73	±	3.08	16.76	±	2.38	15.38	±	2.94	15.29	±	3.63	16.91	±	5.76	12.09	±	5.86	5.78	±	1.17
**Σ**	**72.56**			**51.36**			**130.3**			**79.61**			**85.97**			**77.29**			**124.4**			**142.0**			**101.3**			**34.84**		
**Flavonol-3-O-glycosides**																														
Quercetin-3-O-glucoside	5.86	±	1.16	9.06	±	2.50	21.92	±	1.05	16.70	±	2.27	21.38	±	2.90	10.88	±	2.22	4.98	±	1.03	5.05	±	1.16	1.45	±	0.36	1.36	±	0.34
Quercetin-3-O-arabinoside	11.62	±	1.59	14.87	±	3.80	45.47	±	1.29	27.82	±	1.13	35.65	±	2.67	26.50	±	1.67	7.34	±	1.26	14.48	±	2.77	6.54	±	0.98	3.32	±	0.56
Kaempferol-3-O-rutinoside	n.d.			1.29	±	0.36	8.31	±	0.46	n.d.			n.d.			n.d.			4.08	±	0.89	2.85	±	0.80	1.64	±	0.51	0.21	±	0.07
**Σ**	**17.47**			**25.21**			**75.69**			**44.52**			**57.03**			**37.37**			**16.40**			**22.38**			**9.63**			**4.89**		
**Flavonols**																														
Quercetin	1.76	±	0.61	1.94	±	0.34	2.88	±	0.49	2.59	±	0.18	4.51	±	0.49	1.92	±	0.34	0.91	±	0.07	0.98	±	0.03	0.83	±	0.02	0.75	±	0.01
**Σ**	**1.76**			**1.94**			**2.88**			**2.59**			**4.51**			**1.92**			**0.91**			**0.98**			**0.83**			**0.75**		
***N*-Phenylpropenoyl-L-amino acids**																														
*N*-Coumaroyl-L-aspartate_1	8.62	±	1.13	2.51	±	0.74	14.90	±	3.11	10.48	±	1.70	7.90	±	2.17	13.65	±	2.98	6.50	±	2.37	14.60	±	6.48	7.73	±	2.59	2.40	±	0.24
*N*-Caffeoyl-L-aspartate	5.32	±	3.29	16.22	±	3.48	97.25	±	5.43	104.3	±	7.41	111.5	±	4.36	25.95	±	7.28	69.57	±	18.4	69.68	±	2.72	28.34	±	2.06	15.46	±	4.23
*N*-Coumaroyl-L-aspartate_2	7.63	±	3.47	13.61	±	3.94	64.23	±	5.18	65.90	±	9.64	79.32	±	2.41	28.10	±	5.94	1.13	±	0.07	1.45	±	0.20	3.92	±	0.89	0.58	±	0.05
*N*-Coumaroyl-L-glutamate	0.63	±	0.14	0.59	±	0.12	1.18	±	0.01	1.25	±	0.56	1.23	±	0.18	1.17	±	0.18	0.72	±	0.07	0.36	±	0.09	0.22	±	0.05	0.13	±	0.02
*N*-Feruloyl-L-aspartate	1.93	±	0.66	2.46	±	0.64	5.71	±	0.73	5.88	±	0.72	6.26	±	0.62	3.58	±	0.38	2.15	±	0.32	2.81	±	0.52	1.95	±	0.72	0.91	±	0.36
*N*-Coumaroyl-L-tyrosine	9.45	±	1.11	6.32	±	2.04	23.11	±	1.02	8.99	±	0.60	11.71	±	0.40	17.80	±	0.73	13.65	±	5.11	8.97	±	1.33	6.72	±	0.58	7.09	±	0.19
**Σ**	**33.58**			**41.70**			**206.37**			**196.76**			**217.88**			**90.24**			**93.71**			**97.88**			**48.87**			**26.57**		
**Others**																														
C_11_H_21_O_9_S_1	10.18	±	1.82	3.36	±	1.35	10.66	±	0.39	16.98	±	9.45	21.23	±	3.34	8.14	±	5.76	13.45	±	3.40	12.23	±	2.38	9.20	±	2.79	3.14	±	0.47
C_11_H_21_O_9_S_2	13.90	±	5.38	23.67	±	7.20	18.51	±	3.86	33.54	±	5.27	41.82	±	2.08	14.63	±	5.10	43.67	±	16.8	12.55	±	4.21	22.35	±	7.62	6.54	±	2.71
Hydroxyjasmonic acid sulfate	13.18	±	1.10	21.87	±	9.46	18.53	±	3.84	27.60	±	7.87	35.49	±	11.95	20.76	±	12.01	14.41	±	7.20	11.63	±	5.38	2.88	±	0.98	1.01	±	0.30
Sweroside	4.89	±	1.06	6.01	±	2.91	11.75	±	1.83	22.12	±	12.44	15.83	±	3.72	5.78	±	1.75	7.18	±	1.49	2.15	±	0.39	1.17	±	0.25	1.23	±	0.40
**Σ**	**42.15**			**54.92**			**59.45**			**100.23**			**114.38**			**49.30**			**78.72**			**38.56**			**35.60**			**11.93**		
**Σ Total Flavonoids**	**634.76**			**422.89**			**934.50**			**779.81**			**641.30**			**481.96**			**442.38**			**399.67**			**279.03**			**193.56**		
**Σ Total Tannins**	**365.46**			**216.01**			**561.13**			**399.17**			**363.36**			**299.62**			**341.67**			**306.50**			**217.83**			**121.86**		
**Σ TOTAL mg/kg CBS**	**797.59**			**598.68**			**1379.07**			**1235.18**			**1114.72**			**770.24**			**674.86**			**605.38**			**403.04**			**249.01**		
**Methylxanthines**																														
Theobromine	4.66	±	0.74	5.58	±	0.45	6.12	±	0.41	6.29	±	0.66	5.18	±	0.43	6.53	±	0.76	9.95	±	0.61	9.93	±	0.12	9.80	±	0.38	8.99	±	0.30
Caffeine	0.84	±	0.23	1.77	±	0.29	2.52	±	0.10	2.44	±	0.77	2.70	±	0.11	3.67	±	0.64	5.15	±	0.65	5.80	±	0.18	5.15	±	0.26	4.58	±	0.09
**Σ**	**5.50**			**7.34**			**8.64**			**8.74**			**7.88**			**10.19**			**15.10**			**15.73**			**14.95**			**13.56**		
**TOTAL g/kg CBS**	**5.50**			**7.34**			**8.64**			**8.74**			**7.88**			**10.19**			**15.10**			**15.73**			**14.95**			**13.56**		
**Spectrophotometric assays**																														
TPC (g GAE/kg of CBS)	7.52	±	1.42	9.13	±	0.30	7.41	±	0.35	6.45	±	0.43	7.34	±	0.74	6.36	±	0.47	7.38	±	0.11	7.82	±	0.12	5.88	±	0.35	7.72	±	0.39
TFC (g CE/kg of CBS)	2.53	±	0.78	3.14	±	0.15	3.65	±	0.23	3.30	±	0.94	3.29	±	0.53	2.20	±	0.21	2.56	±	0.18	3.40	±	0.25	1.89	±	0.22	2.51	±	0.18
TTC (g CE/kg of CBS)	1.40	±	0.45	1.59	±	0.06	1.75	±	0.16	1.31	±	0.12	1.30	±	0.15	0.93	±	0.17	1.05	±	0.05	1.34	±	0.08	0.85	±	0.08	1.22	±	0.05
RSA (mmol TE/kg of CBS)	23.22	±	5.09	26.33	±	0.99	23.64	±	1.54	23.35	±	3.87	23.57	±	1.64	21.69	±	2.74	24.26	±	0.74	24.26	±	0.31	17.33	±	1.48	22.92	±	0.86

Results expressed as mean values (*n* = 4) ± standard deviation. n.d. Not detected. The sum (Σ) of each class of chemical compounds is highlighted in bold.

### 3.3. Classification of CBSs Based on Chemical Compounds Determined by HPLC-PDA-MS/MS

**Figure 1:**
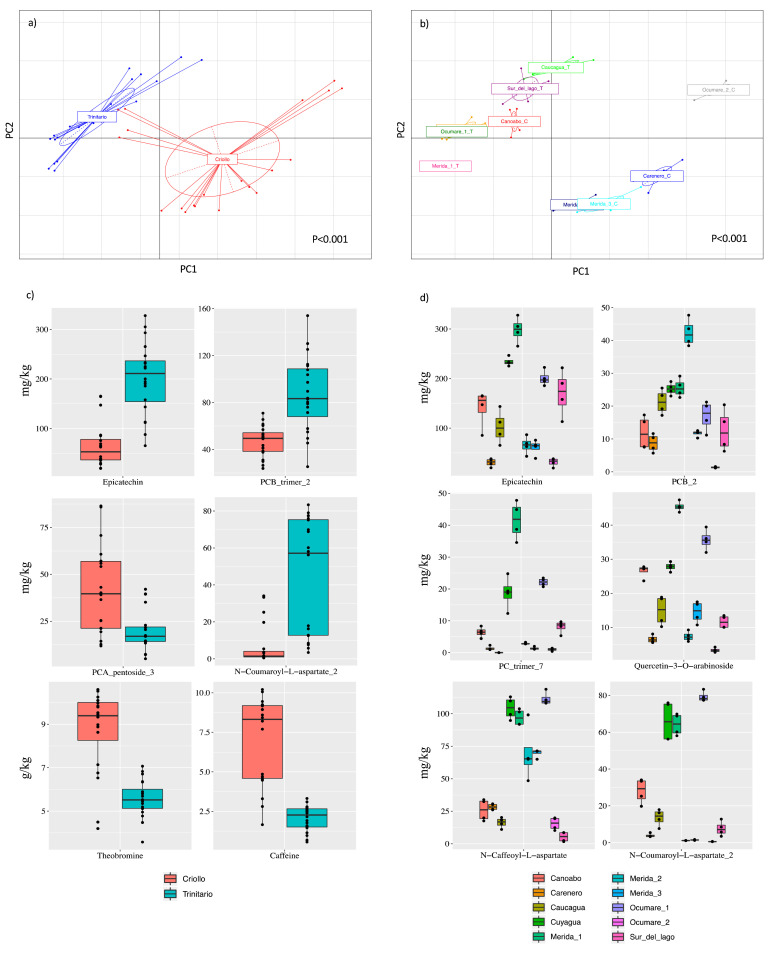
PCA based on the chemical compounds (mg/kg) identified by HPLC-MS/MS in CBS samples from cocoa beans growing in seven geographical areas of Venezuela according to: (**a**) cultivar and (**b**) geographical area of origin. The variance explained by the first component of PCA was 52.14%, while the second component explained 15.16%. Boxplots show the abundance of 12 chemical compounds that can be used as potential markers of CBS (**c**) cultivar and (**d**) geographical origin of cocoa beans.

Based on the chemical profile determined for CBSs yielded from Venezuelan cocoa beans, Figure 1 shows the principal component analysis (PCA) used to find differences among cultivars (Figure 1a) and the geographic areas of Venezuela (Figure 1b).

The PCA explained 67.3% of the total variance and showed a clear separation among the *Criollo* and *Trinitario* cultivars, which was confirmed by the ANOSIM statistical test (*p* < 0.001). The highest content of flavan-3-ols and PCB trimers in the *Criollo* cultivar and the low amounts of *N*-phenylpropenoyl-l-amino acids, compared to *Trinitario* CBSs, allow for a clear separation among cultivars. Several compounds (33 out of the 39) were shown to be significantly different and allowed for the classification of CBSs from Venezuela according to the cultivar (FDR < 0.001 (*n* = 21); FDR < 0.01 (*n* = 8); FDR < 0.05 (*n* = 4), as shown in Figure S3. The boxplots (Figure 1c) show the abundance of the main 12 molecules that allow for the classification of CBSs (FDR < 0.001), considering the most representative key chemical markers described for cocoa products in the literature [13]. Epicatechin, *N*-coumaroyl-l-aspartate, and PCB_trimer_4 were specific compounds with the highest concentrations in *Trinitario* cultivars from different Venezuela geographical areas. On the other hand, the highest concentrations of other cocoa key compounds, such as the methylxanthines theobromine and caffeine, and the polyphenol PCA_pentoside_3, were found in CBS samples of the *Criollo* cultivar. The procyanidins, such as PCB trimer_3 and PCA hexoside_1, were also found to be significant as chemical markers for the Criollo cultivar (Figure S3).

Taking into the account the origin of the CBS, the samples were separated according to the growing area of the respective cocoa beans *(p* < 0.001) (Figure 1b). However, in some cases, an effect of the cultivar was observed on this separation. Despite the samples from the same region being separated and positioned closely in Figure 1b, such as Ocumare and Merida (top for Ocumare and bottom for Merida), the samples remain separated according to their cultivar. Indeed, the samples Merida_2 and Merida_3 (*Criollo*) cluster together, while Merida_1 (*Trinitario*) was separated. The influence of the cultivar on the separation of CBS has been previously reported for the volatile fingerprint of CBS [29]. However, this effect was not perceptible for Canoabo CBSs from the *Criollo* cultivar, which clustered near CBSs from the *Trinitario* cultivar. CBS samples Ocumare_1 and Cuyagua (*Trinitario* cultivar) cluster together due to their similar chemical profile. The geographical proximity of both regions (Figure S1) might be the main factor that contributes to a similar chemical profile as described in the literature for cocoa beans [20]. Together with Merida (Merida_1), these two origins were those with the highest content of chemical compounds determined by HPLC-PDA-MS/MS. CBSs from these regions were also those with the highest content of *N*-phenylpropenoyl-L-amino acids. On the other hand, samples from the *Criollo* cultivar from the Merida (Merida_2, Merida_3) and Carenero regions were clustered closely in the PCA due to the characteristic high content in PCA glycosides. Furthermore, these samples, together with Ocumare_2, were those with the lowest amounts of bioactive compounds. From the HPLC-PDA-MS/MS data, 24 chemical compounds were found as potential markers for the classification of CBSs according to the geographical region (FDR < 0.05), as shown in Figure S4 (see Supplementary Materials). Figure 1d shows the boxplot with the abundance of the most representative key compounds (*n* = 12) that allow for the classification of CBSs (FDR < 0.05) according to the growth region. Protocatechuic acid was found in high concentrations in the CBSs from three close geographical areas: Ocumare, Cayuagua, and Canoabo (Figure S1). On the other hand, CBSs from Caucagua, Sur del Lago, and Canoabo were characterized by the highest amount of epicatechin, one of the main key markers of cocoa. Sample Merida_1 was in the PCA close to the Ocumare_1 and Cayagua due to the key *N*-phenylpropenoyl-l-amino acids, *N*-Caffeoyl-L-aspartate and *N*-coumaroyl-l-aspartate_2, but the presence of high amounts of epicatechin, PCB_trimers isomers 4 and 7, and quercetin-3-*O*-arabinoside allowed for a good discrimination among them. The highest contents of the chemical compounds PCB_2, PCA_pentoside_3, and *N*-Caffeoyl-l-aspartate were found among all CBS samples from Merida, and thus, they might be used as key markers for this region. In the same way, other cocoa key markers, such as the methylxanthines (theobromine and caffeine), allowed the separation of Merida (*Criollo* cultivar) from Carenero and Ocumare_2.

### 3.4. Classification of CBSs Based on Spectrophotometric Analysis Data Set

The spectrophotometric assays dataset that was used to discriminate the CBSs from Venezuela will be discussed in this section.

**Figure 2:**
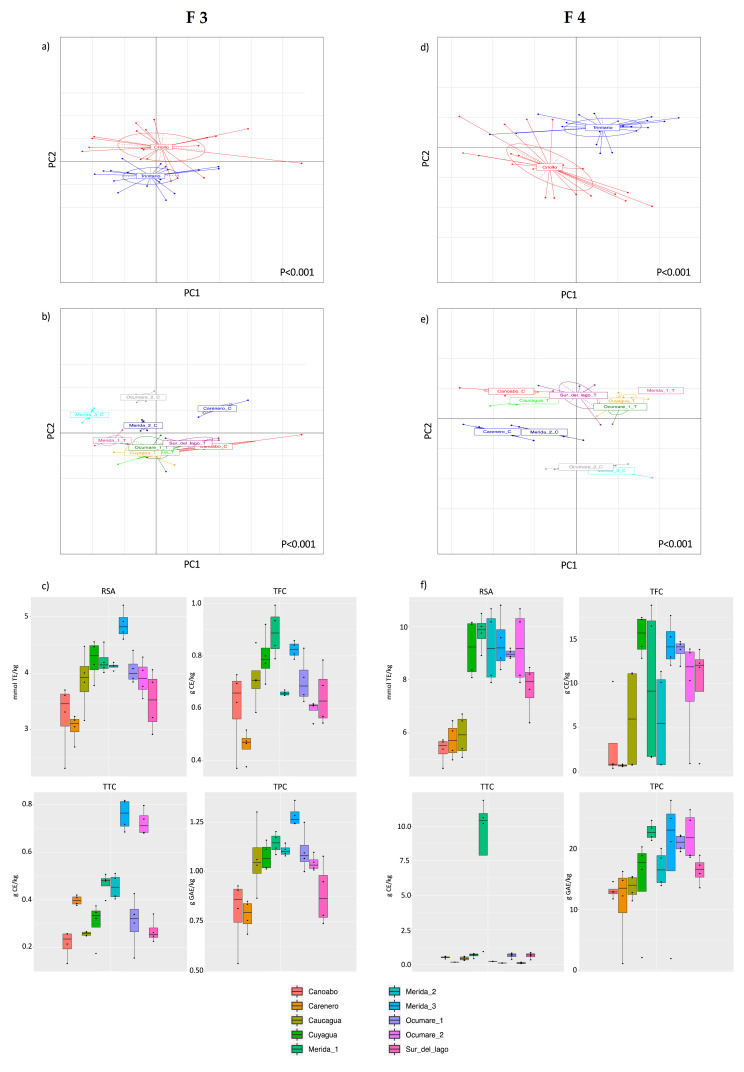
PCAs based on the spectrophotometric assay data (TPC, TFC, TTC, and RSA) of CBS samples from cocoa beans growing in seven geographical areas of Venezuela according to the most representative fractions (F3 and F4). For the F3 PCAs according to (**a**) cultivar and (**b**) geographical area of origin, the variance explained by the first component of PCA was 70.11%, while the second component explained 26.82%. For the F4 PCAs according to (**d**) cultivar and (**e**) geographical area of origin, the variance explained by the first component of PCA was 63.53%, while the second component explained 32.78%. Boxplots show the spectrophotometric assay results that can be used for the classification of CBS according to geographical origin of cocoa beans using F3 (**c**) and F4 (**f**).

Since F3 and F4 were the fractions with a high contribution of bioactive compounds, in Figure 2, the contribution of both fractions for the classification of CBS according to cultivar (Figure 2a (F3) and Figure 2d (F4)) and geographical origin (Figure 2b (F3) and Figure 2e (F4)) is shown.

The PCA of spectrophotometric assay data of fraction F3 and F4 explains 96.93% and 96.31% of the variance, respectively, and shows a clear separation among the two cultivars, *Criollo* and *Trinitario*, confirmed by the ANOSIM statistical test (*p* < 0.001).

Taking into account the geographical regions of Venezuela, a similar pattern of discrimination to that obtained with the HPLC-PDA-MS/MS dataset was achieved for CBS samples (*p* < 0.001) considering fractions F3 (Figure 2b) and F4 (Figure 2e). The spectrophotometric assays confirm the separation of CBSs from Carenero, Merida_2 and Merida_3, and Ocumare_2 from the others in both fractions, and this discrimination was more effective in F3 since in the case of F4, Merida_3 and Ocumare_2 clustered together. On the other hand, F4 showed the best separation of CBSs from Merida_1, Sur del Lago, and Canoabo, similar to that obtained with HPLC-PDA-MS/MS analysis. The boxplot of each spectrophotometric assay result obtained for fractions F3 and F4 is shown in Figure 2c and Figure 2f, respectively. The results highlighted that F3 and F4 datasets allow effective discrimination using the four screening assays, which could be used as a tool for the rapid discrimination of CBS samples from different cultivars and few geographic origins. Fraction F3 Merida_2 was separated due to the high response to the RSA, TTC, and TPC assays, while Carenero and Canoabo samples were on the opposite side of PCA. Likewise, the TFC assay allowed for a complete discrimination of all Merida samples due to their high response to this assay.

### 3.5. Correlation between HPLC-PDA-MS/MS and Spectrophotometric Assays Data Sets

**Figure 3:**
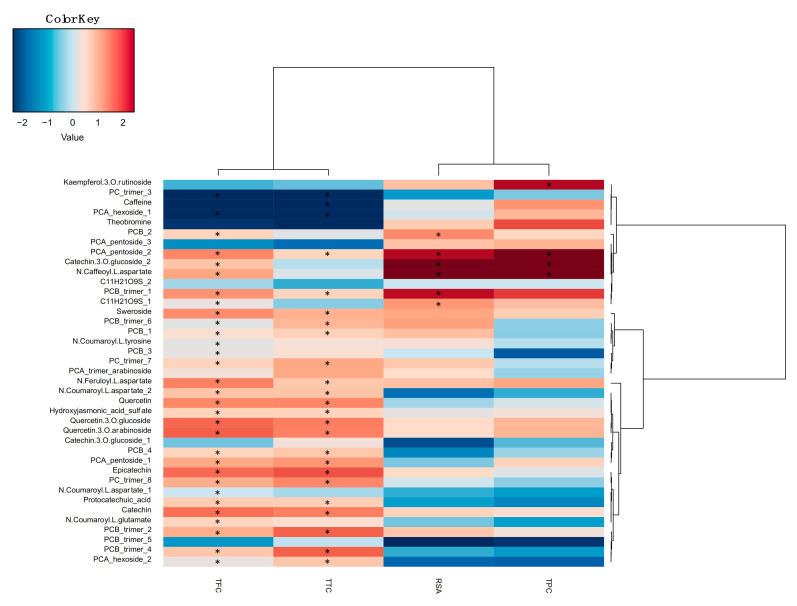
Correlation between the abundance of bioactive compounds and spectrophotometric assay responses. Rows and columns are clustered by Ward linkage hierarchical clustering. The intensity of the colors represents the degree of correlation between the samples and chemical compounds as measured by Spearman’s correlations. Asterisks denote significant correlations after *p* value corrections (FDR < 0.05).

The correlation between spectrophotometric assays and HPLC-PDA-MS/MS showed the relationship between RSA values and polyphenols. The dataset was evaluated separately for each fraction and compared with the respective bioactive compounds determined by chromatographic analysis (Tables S2–S5). The correlation between the analytical techniques for the sum of the fractions F2, F3, and F4 is shown in Figure 3. 

The heatmap clearly shows two main clusters of spectrophotometric assays: TPC and RSA (cluster 1) and TFC and TTC (cluster 2). The high correlation between TPC and RSA has been reported in the literature by several authors, including for CBS samples. Nevertheless, descriptions of the correlation between screening methods and the individual chemical compound are limited [28,36]. In this study, both methods were correlated positively with the chemical compounds, such as PCA_pentoside_3, catechin-3-*O*-glucoside_2, PCB_trimer_1, or *N*-caffeoyl-L-aspartate. Considering the high number of bioactive compounds determined by HPLC-PDA-MS/MS, their correlation with cluster 1 was limited, mainly for the TPC method. The methods TFC and TTC used to determine flavonoids and tannins, respectively, showed a high correlation among them. In addition, a positive correlation between these screening methods and almost all flavonoids identified by chromatography was observed, mainly with flavan-3-ols (catechin and epicatechin) and their derivates. Indeed, significant positive correlations were found between TFC data and several chemical compounds, such as flavan-3-ols monomers (catechin and epicatechin), catechin-3-*O*-glucoside_2, quercetin-3-*O*-glucosides, or the aglycone quercetin. Moreover, *N*-phenylpropenoyl-l-amino acids showed a higher positive correlation with TFC than TTC and a negative correlation with TPC. On the other hand, the TTC method that determines the total amount of tannins showed the highest positive correlation with several procyanidins, mainly type-B trimers (isomers 1, 2, 4, 6, 7, and 8). Moreover, some of these compounds were also correlated positively with antioxidant capacity, such as PCB-trimers 2, 6, and 7 [39]. Other compounds, such as catechin, epicatechin, or *N*-feruloyl-l-aspartate, might also contribute to the antioxidant capacity of the CBS samples, but, despite the positive correlation, this was not significant compared with the other compounds present in CBS and with that described before by other authors [36,39].

## 4. Conclusions

This study provides, for the first time, the chemical fingerprint of cocoa bean shells (CBSs) from Venezuelan cocoa beans, based mainly on methylxanthines and phenolic compounds determined by SPE-HPLC-PDA-ESI-MS/MS. Several compounds, such as catechin glycosides, procyanidins A-type glycosides, and *N*-Feruloyl-l-aspartate, were identified, for the first time, in CBS samples. Moreover, the presence of high amounts of high added-value compounds, such as methylxanthines, *N*-phenylpropenoyl-l-amino acids, flavan-3-ols, and procyanidins, valorizes the byproduct as a food ingredient. CBSs of the *Trinitario* cultivar from the geographical regions of Merida, Cayagua, and Ocumare were those with high contents of bioactive compounds.

The datasets obtained by HPLC-PDA-MS/MS and spectrophotometric assays allowed for the classification of CBSs yielded from cocoa beans from different Venezuelan regions according to both geographical area and cultivar. However, the classification by the screening method (spectrophotometric assays) results required a previous purification process of the extracts and analysis of the fractions separately, which constitutes a critical limitation.

The HPLC-PDA-MS/MS methodology consented to the classification of CBSs according to cultivar and geographical origin through the identification of several chemical compounds recognized as cocoa key markers, such as epicatechin, theobromine, caffeine, *N*-coumaroyl-l-aspartate_2, quercetin-3-*O*-glucoside, PCA-pentoside_3, several procyanidins, etc. The classification of CBSs, according to the cultivar, was also achieved using the ratio of theobromine/caffeine (T/C) that distinguished *Criollo* from *Tinitario* CBSs. The threshold value was established at the value 2 for the CBS differentiation in this study, lower than the value described for cocoa beans in the literature (T/C = 3). This study also proposes, for the first time, the establishment of the epicatechin/catechin ratio (E/C) as a criterium to discriminate CBSs (5 < E/C < 10) from cocoa beans (E/C < 2) and related products, such as chocolate (2 < E/C < 4). This parameter could be of interest for authentication purposes and to control/avoid the adulteration of cocoa powder or other related products by the addition of CBS. 

Finally, this study disclosed a high correlation between spectrophotometric assays, TFC and TTC, and several individual chemical compounds, mainly flavonoids, deter-mined by HPLC-PDA-MS/MS. Instead, the correlation between single compounds and the antioxidant capacity (RSA) was limited to a low number of molecules, and their contribution as antioxidants remains unclear. Despite several new insights of this study, our findings are incomplete and limited to CBSs from Venezuela. Thus, further and exhaustive investigations are necessary to consider a large set of CBS samples from different origins and cultivars to sustain these preliminary findings.

Analogous to cocoa beans, the selective collection and discrimination of this byproduct might be of great interest for the food industry sector, not only for flavor properties, but also because it constitutes an attractive source of bioactive compounds with application as a functional ingredient. Therefore, the valorization, authentication, and selective recovery of CBS might contribute to the sustainability of the cocoa production industry within the circular economy framework.

## Figures and Tables

**Table 1:** Identification of chemical compounds in CBS fraction extracts yielded from roasted cocoa beans from different regions of Venezuela.

Peak	R_t_ (min)	λ_max_	Molecular Formula	Ionization Mode	[M-H] (*m*/*z*)	MS/MS (*m*/*z*)	Tentative Identification	Confirmation/ Ref. ^§^	CBS Fractions			
									F1	F2	F3	F4
1	4.73	259, 293	C_7_H_6_O_4_	[M-H]^−1^	153	109	Protocatechuic acid	[22,30], Std*	-	✓	✓	-
2	6.4	272	C_7_H_8_N_4_O_2_	[M-H]^+1^	181	167, 138, 137	Theobromine	[3,21], Std*	✓	-	-	-
3	6.8	279, 309	C_13_H_13_NO_6_	[M-H]^−1^	278	163	*N*-Coumaroyl-L-aspartate_1	[7,37]	-	✓	-	-
4	7.2	279	C_21_H_24_O_11_	[M-H]^−1^	451	289, 245	Catechin-3-O-glucoside_1	[19]	-	✓	-	-
5	8.13	275	C_11_H_21_O_9_S	[M-H]^−1^	329	241	C_11_H_21_O_9_S_1	[19]	-	✓	-	-
6	8.24	282, 320	C_13_H_13_NO_7_	[M-H]^−1^	294	276, 179, 132	*N*-Caffeoyl-L-aspartate	[7,20,26,37]	-	✓	✓	✓
7	8.63	278	C_15_H_14_O_6_	[M-H]^−1^	289	245, 205, 203, 137, 125	Catechin	[20,22] Std*	-	-	✓	-
8	8.84	278	C_11_H_21_O_9_S	[M-H]^−1^	329	241	C_11_H_21_O_9_S_2	[19]	-	-	✓	✓
9	9.5	279	C_21_H_24_O_11_	[M-H]^−1^	451	289, 245	Catechin-3-O-glucoside_2	[19]	-	✓	-	-
10	9.58	293	C_16_H_22_O_9_	[M-H]^−1^	357	-	Sweroside	[26]	-	-	✓	-
11	9.7	280	C_45_H_38_O_18_	[M-H]^−1^	865	577, 425, 289	Procyanidin B-type trimer_1	[26]	-	-	-	✓
12	10.6	284, 306	C_13_H_13_NO_6_	[M-H]^−1^	278	234, 163, 132	*N*-Coumaroyl-L-aspartate_2	[7,20,26,37]	-	✓	✓	✓
13	11.08	278	C_45_H_38_O_18_	[M-H]^−1^	865	577, 425, 407, 289	Procyanidin B-type trimer_2	[26]	-	-	-	✓
14	11.1	280	C_30_H_26_O_12_	[M-H]^−1^	577	425, 407, 289	Procyanidin B-type_1	[26]	-	✓	✓	✓
15	11.37	280	C_30_H_26_O_12_	[M-H]^−1^	577	425, 407, 289	Procyanidin B-type_2	[26]	-	✓	-	-
16	11.5	278	C_15_H_14_O_6_	[M-H]^−1^	289	245, 205, 203, 137, 125	Epicatechin	[20,22], Std*	-	-	✓	-
17	11.87	286	C_14_H_15_NO_6_	[M-H]^−1^	292	274, 248, 230, 202, 163, 145, 128, 119	*N*-Coumaroyl-L-glutamate	[20,26]	-	-	✓	-
18	12.1	275	C_12_H_18_O_7_S	[M-H]^−1^	305	225	Hydroxyjasmonic acid sulfate	[20]	-	-	✓	-
19	12.2	272	C_8_H_10_N_4_O_2_	[M-H]^+1^	195	181, 151, 138	Caffeine	[3,21], Std*	✓	-	-	-
20	12.25	286, 318	C_14_H_15_NO_7_	[M-H]^−1^	308	276, 264, 246, 193, 149, 134	*N*-Feruloyl-L-aspartate	[7,20,26]	-	-	✓	✓
21	12.78	279	C_45_H_38_O_18_	[M-H]^−1^	865	577, 407, 289	Procyanidin B-type trimer_3	[26]	-	-	-	✓
22	12.84	280	C_30_H_26_O_12_	[M-H]^−1^	577	425, 289	Procyanidin B-type_3	[26]	-	✓	✓	-
23	12.9	280	C_35_H32O_16_	[M-H]^−1^	707	-	Procyanidin A-type pentoside_1	[19]	-	-	✓	-
24	13	278	C_45_H_38_O_18_	[M-H]^−1^	865	577, 425, 407, 289	Procyanidin B-type trimer_4	[26]	-	-	-	✓
25	13.22	280	C_45_H_38_O_18_	[M-H]^−1^	865	577, 289	Procyanidin B-type trimer_5	[26]	-	-	✓	-
26	13.5	280	C_30_H_26_O_12_	[M-H]^−1^	577	289	Procyanidin B-type_4	[26]	-	-	✓	-
27	13.61	279	C_45_H_38_O_18_	[M-H]^−1^	865	577, 289	Procyanidin B-type trimer_6	[26]	-	-	-	✓
28	13.86	280	C_36_H_34_O_17_	[M-H]^−1^	737	611, 449	Procyanidin A-type hexoside _1	[19]	-	-	✓	-
29	14.6	279	C_45_H_38_O_18_	[M-H]^−1^	865	577, 289	Procyanidin B-type trimer_7	[26]	-	-	-	✓
30	14.9	280	C_35_H_32_O_16_	[M-H]^−1^	707	449	Procyanidin A-type pentoside_2	[19]	-	-	✓	✓
31	15.82	279	C_45_H_38_O_18_	[M-H]^−1^	865	577, 425, 407, 289	Procyanidin B-type trimer_8	[26]	-	-	-	✓
32	16.03	279	C_50_H_44_O_22_	[M-H]^−1^	995	865, 407	Procyanidin A-type trimer arabinoside	[20]	-	-	-	✓
33	17.2	278	C_36_H_34_O_17_	[M-H]^−1^	737	611, 539, 449, 289	Procyanidin A-type hexoside_2	[19]	-	-	✓	✓
34	17.5	283	C_18_H_17_NO_5_	[M-H]^−1^	326	282, 206, 163, 145, 134, 119	*N*-Coumaroyl-L-tyrosine (Deoxyclovamide)	[7,20,26,37]	-	-	✓	✓
35	17.7	278	C_35_H_32_O_16_	[M-H]^−1^	707	581, 539, 449, 287	Procyanidin A-type pentoside_3	[19]	-	-	✓	✓
36	18.2	255, 354	C_21_H_20_O_12_	[M-H]^−1^	463	301	Quercetin-3-O-glucoside	[19], Std*	-	-	✓	-
37	18.5	266, 346	C_27_H_30_O_15_	[M-H]^−1^	593	285	Kaempferol-3-O-rutinoside	[20,22]	-	-	✓	-
38	19.4	255, 355	C_20_H_18_O_17_	[M-H]^−1^	433	301	Quercetin-3-O-arabinoside	[19]	-	-	✓	-
39	21.83	275, 365	C_15_H_10_O_7_	[M-H]^−1^	301	-	Quercetin	[22,26], Std*	-	-	-	✓

R_t_—retention time; λ_max_—maximum absorption wavelengths; [M–H]—molecular ions; ✓—indicates the presence of the compound identified; ^§^ Ref—references used to support tentative identification of compounds and Std* (standards available) to confirm the identification. In this work, a total of 39 molecules from different classes of bioactive compounds were identified, such as phenolic acids, flavonoids (including procyanidins, flavan-3-ols, flavonols, and their glycosides), *N*-phenylpropenoyl-L-amino acids, and methylxanthines.

## Supplementary Materials

The following are available online at https://www.mdpi.com/article/10.3390/foods10081791/s1, Figure S1. Fermented and dried cocoa beans used to yield the cocoa bean shell (CBS) samples from different geographical areas in Venezuela, Figure S2. HPLC-PDA-MS/MS chromatograms of a representative CBS raw extract yielded from cocoa beans with origin in Merida and *Criollo* cultivar (Merida_2_C), and its respective fractions yielded by solid phase extraction (SPE), as follows: (a) CBS raw extract; (b) 1st fraction (F1); (c) 2nd fraction (F2); (d) 3rd fraction (F3); and finally, (e) 4th fraction (F4), recorded at 280 nm, Figure S3. Pairwise comparison of several chemical compounds quantified in CBSs by HPLC-PDA-MS/MS that could be used as potential markers among cocoa cultivars. FDR < 0.001; FDR < 0.01 and FDR < 0.05 are highlighted in green, yellow and red, respectively, Figure S4. Pairwise comparison of several chemical compounds quantified in CBSs by HPLC-PDA-MS/MS, which could be used as potential markers among Venezuela geographical regions. FDR < 0.05 are highlighted in red, Table S1. Analytical parameters of HPLC/PDA-MS/MS for the quantification and semi-quantification of polyphenols and methylxanthines in CBS, Table S2. Total amounts of methylxanthines and spectrophotometric assay results determined in fraction F1 yielded from CBS samples from *Trinitario* and *Criollo* cultivars and from different regions of Venezuela. Data are expressed g/kg of CBS, Table S3. Total amounts of bioactive compounds (*n* = 10) and spectrophotometric assay results determined in fraction F2 yielded from CBS samples from *Trinitario* and *Criollo* cultivars and from different regions of Venezuela. Data are expressed as mg/kg of CBS, Table S4. Total amounts of bioactive compounds (*n* = 23) and spectrophotometric assay results determined in fraction F3 yielded from CBS samples from *Trinitario* and *Criollo* cultivars and from different regions of Venezuela. Data are expressed mg/kg of CBS, Table S5. Total amounts of bioactive compounds (*n* = 19) and spectrophotometric assay results determined in fraction F4 yielded from CBS samples from *Trinitario* and *Criollo* cultivars and from different regions of Venezuela. Data are expressed as mg/kg of CBS.
