# Supplementary material for: Characterization and Classification of Cocoa Bean Shells from Different Regions of Venezuela Using HPLC-PDA-MS/MS and Spectrophotometric Techniques Coupled to Chemometric Analysis
Source: Foods. 2021 Aug 2;10(8):1791. doi: 10.3390/foods10081791 (PMC8393802; doi:10.3390/foods10081791)
Supplement: Supplementary file 1 [file foods-10-01791-s001.zip › foods-1229822-supplementary.pdf]

foods

MDPI

Article Supplementary Material

Characterization and Classification of Cocoa Bean Shells from Different Regions of Venezuela Using HPLC-PDA-MS/MS and Spectrophotometric Techniques Coupled to Chemometric Analysis

Letricia Barbosa-Pereira [1,2,*], Simona Belviso [1], Ilario Ferrocino [1], Olga Rojo-Poveda [1,3], and Giuseppe Zeppa [1]

[1] Department of Agriculture, Forestry and Food Sciences (DISAFA), University of Turin, 10095 Grugliasco, Italy; simona.belviso1@posta.istruzione.it (S.B.); ilario.ferrocino@unito.it (I.F.); giuseppe.zeppa@unito.it (G.Z.);

[2] Department of Analytical Chemistry, Nutrition and Food Science, Faculty of Pharmacy, University of Santiago de Compostela, 15782 Santiago de Compostela, Spain; letricia.barbosa.pereira@usc.es (L.B.-P.)

[3] RD3 Department-Unit of Pharmacognosy, Bioanalysis and Drug Discovery, Faculty of Pharmacy, Université libre de Bruxelles, 1050 Brussels, Belgium; Olga.Rojo.Poveda@ulb.be (O.R-P.)

* Correspondence: letricia.barbosa.pereira@usc.es

Abstract: The cocoa bean shell (CBS) is one of the main cocoa byproducts with the prospective to be used as a functional food ingredient due to its nutritional and sensory properties. This study aims to define the chemical fingerprint of CBSs obtained from cocoa beans of diverse cultivars and collected in different geographical areas of Venezuela assessed using high-performance liquid chromatography coupled to photodiodes array and mass spectrometry (HPLC-PDA-MS/MS) and spectrophotometric assays combined with multivariate analysis for classification purposes. The study provides a comprehensive fingerprint and quantitative data for 39 compounds, including methylxanthines and several polyphenols, such as flavan-3-ols, procyanidins and *N*-phenylpropenoyl amino acids. Several key cocoa markers, such as theobromine, epicatechin, quercetin-3-*O*-glucoside, procyanidin_A pentoside_3, and *N*-coumaroyl-L-aspartate_2, were found suitable for the classification of CBS according to their cultivar and origin. Despite the screening methods required a previous purification of the sample, both methodologies appear to be suitable for the classification of CBS with a high correlation between datasets. Finally, preliminary findings on the identification of potential contributors for the radical scavenging activity of CBS were also accomplished to support the valorization of this byproduct as a bioactive ingredient in the production of functional foods.

Keywords: Cocoa bean shell; Fingerprint; Polyphenols; Methylxanthines; HPLC-PDA-ESI-MS/MS; Spectrophotometric screening assays; Principal component analysis; Chemical markers; Traceability; Antioxidant capacity

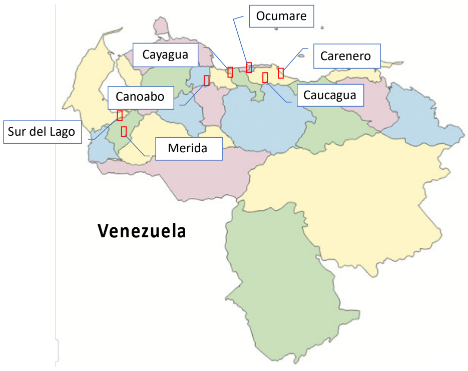

Sample name	Region	Cultivar
Sur del Lago_T	Sur del Lago	*Trinitario*
Caucagua_T	Caucagua	*Trinitario*
Merida_1_T	Merida	*Trinitario*
Cayagua_T	Cuyagua	*Trinitario*
Ocumare_1_T	Ocumare	*Trinitario*
Canoabo_C	Canoabo	*Criollo*
Merida_2_C	Merida	*Criollo*
Merida_3_C	Merida	*Criollo*
Carenero_C	Carenero	*Criollo*
Ocumare_2_C	Ocumare	*Criollo*

Figure S1. Fermented and dried cocoa beans used to yield the cocoa bean shell (CBS) samples from different geographical areas in Venezuela.

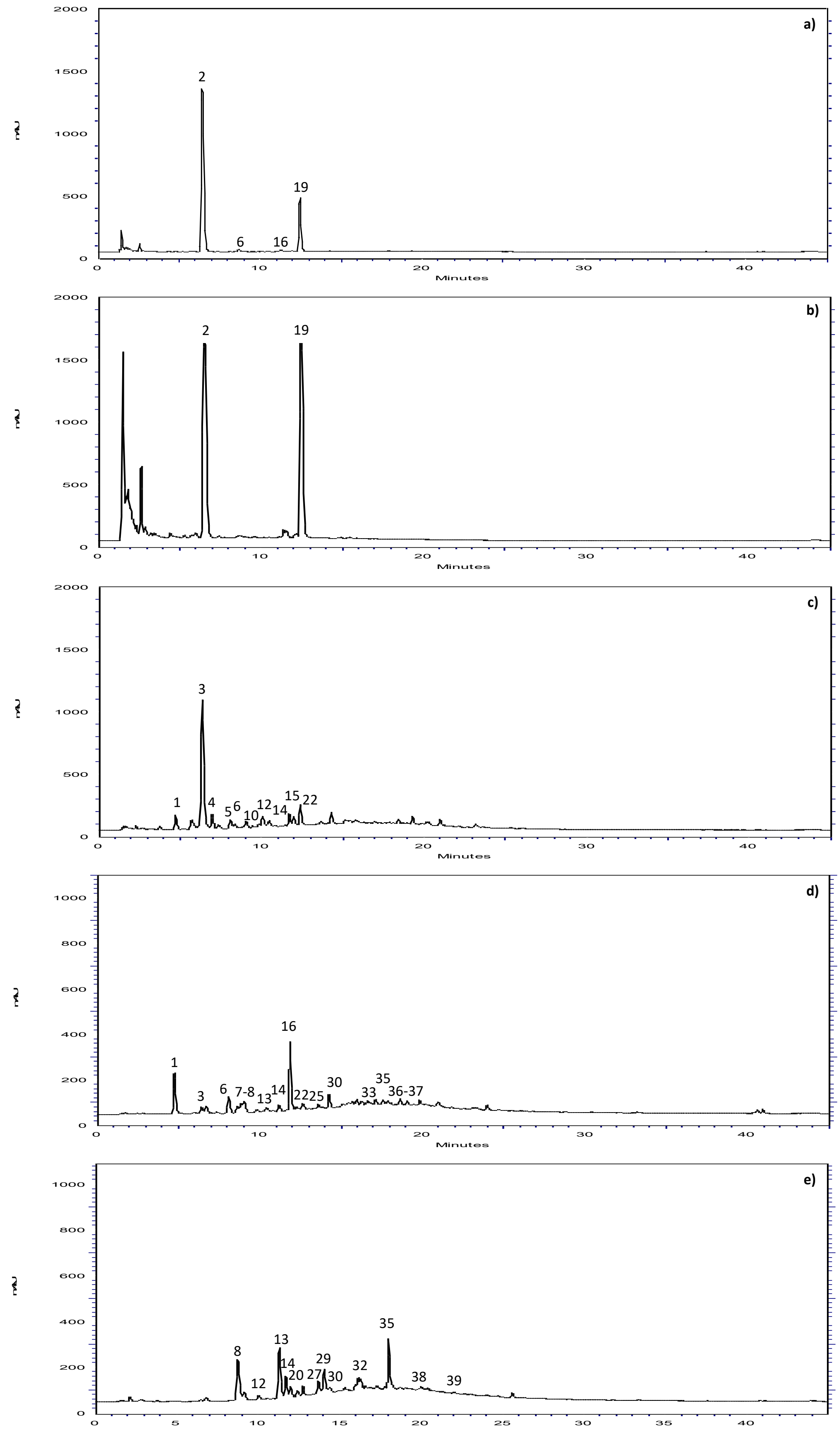

Figure S2. HPLC-PDA-MS/MS chromatograms of a representative CBS raw extract yielded from cocoa beans with origin in Merida and *Criollo* cultivar (Merida_2_C), and its respective fractions yielded by solid phase extraction (SPE), as following: (**a**) CBS raw extract; (**b**) 1st fraction (F1); (**c**) 2nd fraction (F2); (**d**) 3rd fraction (F3); and finally, (**e**) 4th fraction (F4), recorded at 280 nm.

Compound		Criollo
Protocatechuic acid	Trinitario	0.00026
Catechin	Trinitario	6.70E-06
Epicatechin	Trinitario	6.50E-08
PCB_1	Trinitario	0.0051
PCB_2	Trinitario	0.012
PCB_3	Trinitario	0.0031
PCB_4	Trinitario	9.70E-06
PCB_trimer_1	Trinitario	0.0023
PCB_trimer_2	Trinitario	1.30E-05
PC trimer_3	Trinitario	1.10E-06
PCB trimer_4	Trinitario	5.80E-06
PCB trimer_5	Trinitario	0.0045
PCB trimer_6	Trinitario	0.047
PC trimer_7	Trinitario	0.0018
PC trimer_8	Trinitario	0.00012
PCA pentoside_1	Trinitario	6.60E-06
PCA pentoside_3	Trinitario	0.00093
PCA hexoside_1	Trinitario	1.10E-06
PCA hexoside_2	Trinitario	6.00E-04
PCA trimer arabinoside	Trinitario	0.0043
Catechin-3-O-glucoside_1	Trinitario	7.40E-05
Catechin-3-O-glucoside_2	Trinitario	0.021
Quercetin-3-O-glucoside	Trinitario	2.40E-06
Quercetin-3-O-arabinoside	Trinitario	1.00E-04
Quercetin	Trinitario	9.00E-07
C11H21O9S_2	Trinitario	0.043
Hydroxyjasmonic acid sulfate	Trinitario	0.00023
Sweroside	Trinitario	3.70E-05
N-Coumaroyl-L-aspartate_2	Trinitario	7.60E-06
N-Feruloyl-L-aspartate	Trinitario	0.0021
N-Coumaroyl-L-glutamate	Trinitario	0.0023
Theobromine	Trinitario	2.00E-05
Caffeine	Trinitario	6.90E-07

Figure S3. Pairwise comparison of several chemical compounds quantified in CBSs by HPLC-PDA-MS/MS that could be used as potential markers among cocoa cultivars. FDR < 0.001; FDR < 0.01 and FDR < 0.05 are highlighted in green, yellow and red, respectively.

Protocatechuic acid

	Canoabo_C	Carenero_C	Caucagua_T	Cuyagua_T	Merida_1_T	Merida_2_C	Merida_3_C	Ocumare_1_T	Ocumare_2_C
Carenero_C	0.04	-	-	-	-	-	-	-	-
Caucagua_T	0.04	0.04	-	-	-	-	-	-	-
Cuyagua_T	0.91	0.04	0.04	-	-	-	-	-	-
Merida_1_T	1.00	0.04	0.04	0.74	-	-	-	-	-
Merida_2_C	0.04	0.15	0.40	0.04	0.04	-	-	-	-
Merida_3_C	0.04	0.04	0.53	0.04	0.04	0.91	-	-	-
Ocumare_1_T	0.24	0.04	0.15	0.15	0.24	0.04	0.08	-	-
Ocumare_2_C	0.04	0.04	0.04	0.04	0.04	0.04	0.04	0.04	-
Sur_del_lago_T	0.04	0.04	0.24	0.04	0.08	0.04	0.04	0.53	0.04

Catechin

	Canoabo_C	Carenero_C	Caucagua_T	Cuyagua_T	Merida_1_T	Merida_2_C	Merida_3_C	Ocumare_1_T	Ocumare_2_C
Carenero_C	0.041	-	-	-	-	-	-	-	-
Caucagua_T	0.225	0.041	-	-	-	-	-	-	-
Cuyagua_T	0.041	0.041	0.041	-	-	-	-	-	-
Merida_1_T	0.041	0.041	0.041	1	-	-	-	-	-
Merida_2_C	0.072	0.041	0.072	0.041	0.041	-	-	-	-
Merida_3_C	0.072	0.072	0.221	0.041	0.041	0.906	-	-	-
Ocumare_1_T	0.041	0.041	0.041	0.041	0.041	0.041	0.041	-	-
Ocumare_2_C	0.041	0.367	0.041	0.041	0.041	0.041	0.041	0.041	-
Sur_del_lago_T	0.367	0.041	0.906	0.041	0.041	0.072	0.221	0.041	0.041

Epicatechin

	Canoabo_C	Carenero_C	Caucagua_T	Cuyagua_T	Merida_1_T	Merida_2_C	Merida_3_C	Ocumare_1_T	Ocumare_2_C
Carenero_C	0.036	-	-	-	-	-	-	-	-
Caucagua_T	0.22	0.036	-	-	-	-	-	-	-
Cuyagua_T	0.036	0.036	0.036	-	-	-	-	-	-
Merida_1_T	0.036	0.036	0.036	0.036	-	-	-	-	-
Merida_2_C	0.068	0.036	0.129	0.036	0.036	-	-	-	-
Merida_3_C	0.036	0.036	0.129	0.036	0.036	0.906	-	-	-
Ocumare_1_T	0.036	0.036	0.036	0.036	0.036	0.036	0.036	-	-
Ocumare_2_C	0.036	1	0.036	0.036	0.036	0.036	0.036	0.036	-
Sur_del_lago_T	0.508	0.036	0.068	0.036	0.036	0.036	0.036	0.367	0.036

PCB_2

	Canoabo_C	Carenero_C	Caucagua_T	Cuyagua_T	Merida_1_T	Merida_2_C	Merida_3_C	Ocumare_1_T	Ocumare_2_C
Carenero_C	0.386	-	-	-	-	-	-	-	-
Caucagua_T	0.083	0.044	-	-	-	-	-	-	-
Cuyagua_T	0.044	0.044	0.243	-	-	-	-	-	-
Merida_1_T	0.044	0.044	0.243	1	-	-	-	-	-
Merida_2_C	0.044	0.044	0.044	0.044	0.044	-	-	-	-
Merida_3_C	1	0.156	0.044	0.044	0.044	0.044	-	-	-
Ocumare_1_T	0.243	0.083	0.386	0.044	0.044	0.044	0.243	-	-
Ocumare_2_C	0.044	0.044	0.044	0.044	0.044	0.044	0.044	0.044	-
Sur_del_lago_T	0.949	0.533	0.156	0.044	0.044	0.044	1	0.386	0.044

PCB_3

	Canoabo_C	Carenero_C	Caucagua_T	Cuyagua_T	Merida_1_T	Merida_2_C	Merida_3_C	Ocumare_1_T	Ocumare_2_C
Carenero_C	0.151	-	-	-	-	-	-	-	-
Caucagua_T	0.151	0.735	-	-	-	-	-	-	-
Cuyagua_T	0.046	0.046	0.046	-	-	-	-	-	-
Merida_1_T	0.443	0.046	0.046	0.046	-	-	-	-	-
Merida_2_C	0.735	0.046	0.046	0.046	0.417	-	-	-	-
Merida_3_C	0.151	0.417	0.807	0.046	0.046	0.046	-	-	-
Ocumare_1_T	1	0.083	0.083	0.046	0.046	0.046	0.083	-	-
Ocumare_2_C	0.046	0.046	0.046	0.046	0.046	0.046	0.046	0.046	-
Sur_del_lago_T	0.417	0.046	0.046	0.443	1	0.735	0.046	0.046	0.046

PCB_trimer_4

	Canoabo_C	Carenero_C	Caucagua_T	Cuyagua_T	Merida_1_T	Merida_2_C	Merida_3_C	Ocumare_1_T	Ocumare_2_C
Carenero_C	0.041	-	-	-	-	-	-	-	-
Caucagua_T	0.076	0.041	-	-	-	-	-	-	-
Cuyagua_T	0.386	0.041	0.041	-	-	-	-	-	-
Merida_1_T	0.041	0.041	0.041	0.041	-	-	-	-	-
Merida_2_C	0.041	0.663	0.041	0.041	0.041	-	-	-	-
Merida_3_C	0.041	0.363	0.041	0.041	0.041	0.663	-	-	-
Ocumare_1_T	0.386	0.041	0.041	0.243	0.041	0.041	0.041	-	-
Ocumare_2_C	0.041	0.663	0.041	0.041	0.041	0.513	0.139	0.041	-
Sur_del_lago_T	0.076	0.041	0.041	0.52	0.143	0.041	0.041	0.041	0.041

PCB_trimer_5

	Canoabo_C	Carenero_C	Caucagua_T	Cuyagua_T	Merida_1_T	Merida_2_C	Merida_3_C	Ocumare_1_T	Ocumare_2_C
Carenero_C	0.046	-	-	-	-	-	-	-	-
Caucagua_T	0.046	0.046	-	-	-	-	-	-	-
Cuyagua_T	0.156	0.046	0.046	-	-	-	-	-	-
Merida_1_T	0.25	0.046	0.046	0.52	-	-	-	-	-
Merida_2_C	0.25	0.046	0.406	0.046	0.046	-	-	-	-
Merida_3_C	0.046	0.52	0.046	0.406	0.046	0.046	-	-	-
Ocumare_1_T	0.701	0.046	0.046	0.086	0.086	0.194	0.046	-	-
Ocumare_2_C	0.046	0.772	0.046	0.046	0.046	0.046	0.52	0.046	-
Sur_del_lago_T	0.156	0.046	0.52	0.046	0.046	0.701	0.046	0.156	0.046

PC_trimer_7

	Canoabo_C	Carenero_C	Caucagua_T	Cuyagua_T	Merida_1_T	Merida_2_C	Merida_3_C	Ocumare_1_T	Ocumare_2_C
Carenero_C	0.033	-	-	-	-	-	-	-	-
Caucagua_T	0.033	0.033	-	-	-	-	-	-	-
Cuyagua_T	0.033	0.033	0.033	-	-	-	-	-	-
Merida_1_T	0.033	0.033	0.033	0.033	-	-	-	-	-
Merida_2_C	0.033	0.033	0.033	0.033	0.033	-	-	-	-
Merida_3_C	0.033	0.678	0.033	0.033	0.033	0.033	-	-	-
Ocumare_1_T	0.033	0.033	0.033	0.367	0.033	0.033	0.033	-	-
Ocumare_2_C	0.033	0.686	0.033	0.033	0.033	0.033	0.49	0.033	-
Sur_del_lago_T	0.367	0.033	0.033	0.033	0.033	0.033	0.033	0.033	0.033

PCA pentoside_3

	Canoabo_C	Carenero_C	Caucagua_T	Cuyagua_T	Merida_1_T	Merida_2_C	Merida_3_C	Ocumare_1_T	Ocumare_2_C
Carenero_C	0.043	-	-	-	-	-	-	-	-
Caucagua_T	0.043	0.043	-	-	-	-	-	-	-
Cuyagua_T	0.367	0.043	0.043	-	-	-	-	-	-
Merida_1_T	0.043	0.367	0.043	0.043	-	-	-	-	-
Merida_2_C	0.043	0.701	0.043	0.043	0.237	-	-	-	-
Merida_3_C	0.043	0.043	0.043	0.043	0.043	0.367	-	-	-
Ocumare_1_T	0.083	0.043	0.043	1	0.043	0.043	0.043	-	-
Ocumare_2_C	0.147	0.043	0.147	0.237	0.043	0.043	0.043	0.367	-
Sur_del_lago_T	0.043	0.043	0.147	0.237	0.043	0.043	0.043	0.147	0.701

PCA hexoside_1

	Canoabo_C	Carenero_C	Caucagua_T	Cuyagua_T	Merida_1_T	Merida_2_C	Merida_3_C	Ocumare_1_T	Ocumare_2_C
Carenero_C	0.026	-	-	-	-	-	-	-	-
Caucagua_T	-	0.026	-	-	-	-	-	-	-
Cuyagua_T	-	0.026	-	-	-	-	-	-	-
Merida_1_T	-	0.026	-	-	-	-	-	-	-
Merida_2_C	0.026	0.032	0.026	0.026	0.026	-	-	-	-
Merida_3_C	0.026	0.343	0.026	0.026	0.026	0.059	-	-	-
Ocumare_1_T	-	0.026	-	-	-	0.026	0.026	-	-
Ocumare_2_C	0.026	0.032	0.026	0.026	0.026	0.032	0.032	0.026	-
Sur_del_lago_T	-	0.026	-	-	-	0.026	0.026	-	0.026

PCA hexoside_2

	Canoabo_C	Carenero_C	Caucagua_T	Cuyagua_T	Merida_1_T	Merida_2_C	Merida_3_C	Ocumare_1_T	Ocumare_2_C
Carenero_C	0.043	-	-	-	-	-	-	-	-
Caucagua_T	0.078	0.396	-	-	-	-	-	-	-
Cuyagua_T	0.52	0.043	0.151	-	-	-	-	-	-
Merida_1_T	0.043	0.043	0.043	0.043	-	-	-	-	-
Merida_2_C	0.043	0.886	0.52	0.043	0.043	-	-	-	-
Merida_3_C	0.043	0.043	0.886	0.043	0.043	0.243	-	-	-
Ocumare_1_T	0.886	0.043	0.043	0.396	0.043	0.043	0.043	-	-
Ocumare_2_C	0.043	0.043	0.043	0.043	0.043	0.043	0.043	0.043	-
Sur_del_lago_T	0.243	0.043	0.243	0.52	0.043	0.078	0.043	0.078	0.043

Catechin-3-O-glucoside_2

	Canoabo_C	Carenero_C	Caucagua_T	Cuyagua_T	Merida_1_T	Merida_2_C	Merida_3_C	Ocumare_1_T	Ocumare_2_C
Carenero_C	0.04	-	-	-	-	-	-	-	-
Caucagua_T	0.04	0.41	-	-	-	-	-	-	-
Cuyagua_T	0.04	0.04	0.04	-	-	-	-	-	-
Merida_1_T	0.04	0.41	1	0.04	-	-	-	-	-
Merida_2_C	0.04	0.04	0.04	1	0.04	-	-	-	-
Merida_3_C	0.04	0.04	0.41	0.04	0.04	0.04	-	-	-
Ocumare_1_T	0.04	0.04	0.04	0.72	0.04	0.55	0.04	-	-
Ocumare_2_C	0.04	0.72	0.2	0.04	0.04	0.04	0.04	0.04	-
Sur_del_lago_T	0.04	0.26	0.72	0.04	0.55	0.04	0.41	0.04	0.04

Quercetin-3-O-glucoside

	Canoabo_C	Carenero_C	Caucagua_T	Cuyagua_T	Merida_1_T	Merida_2_C	Merida_3_C	Ocumare_1_T	Ocumare_2_C
Carenero_C	0.038	-	-	-	-	-	-	-	-
Caucagua_T	0.508	0.038	-	-	-	-	-	-	-
Cuyagua_T	0.038	0.038	0.038	-	-	-	-	-	-
Merida_1_T	0.038	0.038	0.038	0.038	-	-	-	-	-
Merida_2_C	0.038	0.038	0.135	0.038	0.038	-	-	-	-
Merida_3_C	0.038	0.038	0.135	0.038	0.038	1	-	-	-
Ocumare_1_T	0.038	0.038	0.038	0.135	1	0.038	0.038	-	-
Ocumare_2_C	0.038	0.508	0.038	0.038	0.038	0.038	0.038	0.038	-
Sur_del_lago_T	0.073	0.038	0.231	0.038	0.038	0.386	0.508	0.038	0.038

Quercetin-3-O-arabinoside

	Canoabo_C	Carenero_C	Caucagua_T	Cuyagua_T	Merida_1_T	Merida_2_C	Merida_3_C	Ocumare_1_T	Ocumare_2_C
Carenero_C	0.033	-	-	-	-	-	-	-	-
Caucagua_T	0.033	0.033	-	-	-	-	-	-	-
Cuyagua_T	0.359	0.033	0.033	-	-	-	-	-	-
Merida_1_T	0.033	0.033	0.033	0.033	-	-	-	-	-
Merida_2_C	0.033	0.497	0.033	0.033	0.033	-	-	-	-
Merida_3_C	0.033	0.033	0.886	0.033	0.033	0.033	-	-	-
Ocumare_1_T	0.033	0.033	0.033	0.033	0.033	0.033	0.033	-	-
Ocumare_2_C	0.033	0.033	0.033	0.033	0.033	0.033	0.033	0.033	-
Sur_del_lago_T	0.033	0.033	0.332	0.033	0.033	0.033	0.21	0.033	0.033

Kaempferol-3-O-rutinoside

	Canoabo_C	Carenero_C	Caucagua_T	Cuyagua_T	Merida_1_T	Merida_2_C	Merida_3_C	Ocumare_1_T	Ocumare_2_C
Carenero_C	0.031	-	-	-	-	-	-	-	-
Caucagua_T	0.031	0.2	-	-	-	-	-	-	-
Cuyagua_T	-	0.031	0.031	-	-	-	-	-	-
Merida_1_T	0.031	0.031	0.031	0.031	-	-	-	-	-
Merida_2_C	0.031	0.031	0.031	0.031	0.031	-	-	-	-
Merida_3_C	0.031	0.117	0.031	0.031	0.031	0.117	-	-	-
Ocumare_1_T	-	0.031	0.031	-	0.031	0.031	0.031	-	-
Ocumare_2_C	0.031	0.031	0.031	0.031	0.031	0.031	0.031	0.031	-
Sur_del_lago_T	-	0.031	0.031	-	0.031	0.031	0.031	-	0.031

Quercetin

	Canoabo_C	Carenero_C	Caucagua_T	Cuyagua_T	Merida_1_T	Merida_2_C	Merida_3_C	Ocumare_1_T	Ocumare_2_C
Carenero_C	0.04	-	-	-	-	-	-	-	-
Caucagua_T	0.886	0.04	-	-	-	-	-	-	-
Cuyagua_T	0.066	0.04	0.066	-	-	-	-	-	-
Merida_1_T	0.066	0.04	0.066	0.367	-	-	-	-	-
Merida_2_C	0.04	0.066	0.04	0.04	0.04	-	-	-	-
Merida_3_C	0.04	0.04	0.04	0.04	0.04	0.263	-	-	-
Ocumare_1_T	0.04	0.04	0.04	0.04	0.04	0.04	0.04	-	-
Ocumare_2_C	0.04	0.04	0.04	0.04	0.04	0.04	0.04	0.04	-
Sur_del_lago_T	0.886	0.04	0.718	0.225	0.066	0.04	0.04	0.04	0.04

Sweroside

	Canoabo_C	Carenero_C	Caucagua_T	Cuyagua_T	Merida_1_T	Merida_2_C	Merida_3_C	Ocumare_1_T	Ocumare_2_C
Carenero_C	0.044	-	-	-	-	-	-	-	-
Caucagua_T	1	0.044	-	-	-	-	-	-	-
Cuyagua_T	0.044	0.044	0.076	-	-	-	-	-	-
Merida_1_T	0.044	0.044	0.076	0.735	-	-	-	-	-
Merida_2_C	0.735	0.044	0.735	0.076	0.076	-	-	-	-
Merida_3_C	0.044	0.044	0.143	0.044	0.044	0.044	-	-	-
Ocumare_1_T	0.044	0.044	0.044	1	0.243	0.044	0.044	-	-
Ocumare_2_C	0.044	0.806	0.044	0.044	0.044	0.044	0.044	0.044	-
Sur_del_lago_T	0.575	0.044	0.735	0.044	0.044	0.143	0.044	0.044	0.044

N-Caffeoyl-L-aspartate

	Canoabo_C	Carenero_C	Caucagua_T	Cuyagua_T	Merida_1_T	Merida_2_C	Merida_3_C	Ocumare_1_T	Ocumare_2_C
Carenero_C	1	-	-	-	-	-	-	-	-
Caucagua_T	0.225	0.036	-	-	-	-	-	-	-
Cuyagua_T	0.036	0.036	0.036	-	-	-	-	-	-
Merida_1_T	0.036	0.036	0.036	0.376	-	-	-	-	-
Merida_2_C	0.036	0.036	0.036	0.069	0.135	-	-	-	-
Merida_3_C	0.036	0.036	0.036	0.036	0.036	0.508	-	-	-
Ocumare_1_T	0.036	0.036	0.036	0.508	0.036	0.036	0.036	-	-
Ocumare_2_C	0.169	0.036	0.906	0.036	0.036	0.036	0.036	0.036	-
Sur_del_lago_T	0.036	0.036	0.036	0.036	0.036	0.036	0.036	0.036	0.036

N-Coumaroyl-L-aspartate_2

	Canoabo_C	Carenero_C	Caucagua_T	Cuyagua_T	Merida_1_T	Merida_2_C	Merida_3_C	Ocumare_1_T	Ocumare_2_C
Carenero_C	0.031	-	-	-	-	-	-	-	-
Caucagua_T	0.031	0.031	-	-	-	-	-	-	-
Cuyagua_T	0.031	0.031	0.031	-	-	-	-	-	-
Merida_1_T	0.031	0.031	0.031	1	-	-	-	-	-
Merida_2_C	0.031	0.031	0.031	0.031	0.031	-	-	-	-
Merida_3_C	0.031	0.031	0.031	0.031	0.031	0.087	-	-	-
Ocumare_1_T	0.031	0.031	0.031	0.031	0.031	0.031	0.031	-	-
Ocumare_2_C	0.031	0.031	0.031	0.031	0.031	0.031	0.031	0.031	-
Sur_del_lago_T	0.031	0.12	0.205	0.031	0.031	0.031	0.031	0.031	0.031

N-Feruloyl-L-aspartate

	Canoabo_C	Carenero_C	Caucagua_T	Cuyagua_T	Merida_1_T	Merida_2_C	Merida_3_C	Ocumare_1_T	Ocumare_2_C
Carenero_C	0.046	-	-	-	-	-	-	-	-
Caucagua_T	0.089	0.383	-	-	-	-	-	-	-
Cuyagua_T	0.046	0.046	0.046	-	-	-	-	-	-
Merida_1_T	0.046	0.046	0.046	0.927	-	-	-	-	-
Merida_2_C	0.046	1	0.735	0.046	0.046	-	-	-	-
Merida_3_C	0.273	0.383	0.56	0.046	0.046	0.325	-	-	-
Ocumare_1_T	0.046	0.046	0.046	0.735	0.417	0.046	0.046	-	-
Ocumare_2_C	0.046	0.161	0.046	0.046	0.046	0.046	0.046	0.046	-
Sur_del_lago_T	0.046	1	0.56	0.046	0.046	0.735	0.161	0.046	0.161

N-Coumaroyl-L-tyrosine

	Canoabo_C	Carenero_C	Caucagua_T	Cuyagua_T	Merida_1_T	Merida_2_C	Merida_3_C	Ocumare_1_T	Ocumare_2_C
Carenero_C	0.043	-	-	-	-	-	-	-	-
Caucagua_T	0.043	0.927	-	-	-	-	-	-	-
Cuyagua_T	0.043	0.043	0.151	-	-	-	-	-	-
Merida_1_T	0.043	0.043	0.043	0.043	-	-	-	-	-
Merida_2_C	0.417	0.043	0.083	0.257	0.043	-	-	-	-
Merida_3_C	0.043	0.043	0.151	1	0.043	0.417	-	-	-
Ocumare_1_T	0.043	0.043	0.043	0.043	0.043	1	0.043	-	-
Ocumare_2_C	0.043	0.546	0.927	0.043	0.043	0.043	0.043	0.043	-
Sur_del_lago_T	0.043	0.043	0.151	0.753	0.043	0.546	0.546	0.043	0.043

N-Coumaroyl-L-glutamate

	Canoabo_C	Carenero_C	Caucagua_T	Cuyagua_T	Merida_1_T	Merida_2_C	Merida_3_C	Ocumare_1_T	Ocumare_2_C
Carenero_C	0.044	-	-	-	-	-	-	-	-
Caucagua_T	0.044	0.044	-	-	-	-	-	-	-
Cuyagua_T	1	0.044	0.151	-	-	-	-	-	-
Merida_1_T	0.376	0.044	0.044	1	-	-	-	-	-
Merida_2_C	0.044	0.044	0.188	0.406	0.044	-	-	-	-
Merida_3_C	0.044	0.25	0.044	0.044	0.044	0.044	-	-	-
Ocumare_1_T	0.771	0.044	0.044	1	1	0.044	0.044	-	-
Ocumare_2_C	0.044	0.08	0.044	0.044	0.044	0.044	0.044	0.044	-
Sur_del_lago_T	0.044	0.044	0.972	0.151	0.044	0.648	0.08	0.044	0.044

Theobromine

	Canoabo_C	Carenero_C	Caucagua_T	Cuyagua_T	Merida_1_T	Merida_2_C	Merida_3_C	Ocumare_1_T	Ocumare_2_C
Carenero_C	0.049	-	-	-	-	-	-	-	-
Caucagua_T	1	0.049	-	-	-	-	-	-	-
Cuyagua_T	0.441	0.049	0.166	-	-	-	-	-	-
Merida_1_T	0.972	0.049	0.265	0.791	-	-	-	-	-
Merida_2_C	0.049	1	0.049	0.049	0.049	-	-	-	-
Merida_3_C	0.049	0.972	0.049	0.049	0.049	1	-	-	-
Ocumare_1_T	1	0.049	0.265	0.166	0.166	0.049	0.049	-	-
Ocumare_2_C	0.049	0.092	0.049	0.049	0.049	0.575	0.049	0.049	-
Sur_del_lago_T	0.575	0.049	0.265	0.092	0.049	0.049	0.049	0.575	0.049

Caffeine

	Canoabo_C	Carenero_C	Caucagua_T	Cuyagua_T	Merida_1_T	Merida_2_C	Merida_3_C	Ocumare_1_T	Ocumare_2_C
Carenero_C	0.039	-	-	-	-	-	-	-	-
Caucagua_T	0.143	0.039	-	-	-	-	-	-	-
Cuyagua_T	0.718	0.039	0.376	-	-	-	-	-	-
Merida_1_T	0.376	0.039	0.039	1	-	-	-	-	-
Merida_2_C	0.039	0.376	0.039	0.039	0.039	-	-	-	-
Merida_3_C	0.039	0.056	0.039	0.039	0.039	0.52	-	-	-
Ocumare_1_T	0.376	0.039	0.039	1	0.143	0.039	0.039	-	-
Ocumare_2_C	0.376	0.039	0.039	0.039	0.039	0.039	0.039	0.039	-
Sur_del_lago_T	0.039	0.039	0.039	0.039	0.039	0.039	0.039	0.039	0.039

Figure S4. Pairwise comparison of several chemical compounds quantified in CBSs by HPLC-PDA-MS/MS, which could be used as potential markers among Venezuela geographical regions. FDR < 0.05 are highlighted in red.

Table S1. Analytical parameters of HPLC/PDA-MS/MS for the quantification and semi-quantification of polyphenols and methylxanthines in CBS.

Copyright: © 2021 by the authors. Licensee MDPI, Basel, Switzerland. This article is an open access article distributed under the terms and conditions of the Creative Commons Attribution (CC BY) license (http://creativecommons.org/licenses/by/4.0/).

**Compound**	**λ_{max}**	**Calibration Curve**	**Linearity (R^2)**	**Linear Working Range (mg/L)**	**LOD (mg/L)**	**LOQ (mg/L)**	**RSD (%)**
Protocatechuic acid	293	y = 78697x + 19672	0.9997	1.01–101	0.05	0.20	0.98
Theobromine	272	y = 132387x + 50067	0.9995	0.51–102	0.05	0.26	2.45
Procyanind B1	280	y = 27278x - 5153.4	0.9998	1.00–100	0.50	1.00	1.00
Catechin	278	y = 32326x - 13902	0.9999	0.49–97.0	0.20	0.49	1.03
Procyanind B2	273	y = 18790x - 12454	0.9999	1.00–100	0.50	1.00	1.08
Epicatechin	278	y = 31724x + 35810	0.9998	0.99–198	0.50	0.20	0.69
Caffeine	272	y = 117797x + 41653	0.9996	0.49–98.0	0.05	0.25	1.14
Quercetin-3-O-glucoside	354	y = 84895x + 14596	0.9996	0.60–110	0.10	0.60	1.40
Quercetin	365	y = 141529x - 121124	0.9989	1.20–57.5	0.06	0.12	1.77
Caffeic acid	325	y = 273358x - 32405	0.9999	0.50–20.0	0.10	0.20	2.50
p-Coumaric acid	325	y = 180057x + 5449.6	0.9996	0.23–56.5	0.11	0.23	2.99
Ferulic acid	325	y = 276968x + 30964	0.9998	0.25–20.0	0.05	0.10	1.25

R^2 – Coefficient of determination; LOD – Limit of determination; LOQ – Limit of quantification.

Table S2. Total amounts of methylxanthines and spectrophotometric assays results determined in fraction F1 yielded from CBS samples from *Trinitario* and *Criollo* cultivars and from different regions of Venezuela. Data are expressed g/kg of CBS.

Compound	Sur del Lago_T	Caucagua_T	Merida_1_T	Cuyagua_T	Ocumare_1_T	Canoabo_C	Merida_2_C	Merida_3_C	Carenero_C	Ocumare_2_C
**Methylxanthines**										
Theobromine	4.66 ± 0.74	5.58 ± 0.45	6.12 ± 0.41	6.29 ± 0.66	5.18 ± 0.43	6.53 ± 0.76	9.95 ± 0.61	9.93 ± 0.12	9.80 ± 0.38	8.99 ± 0.30
Caffeine	0.84 ± 0.23	1.77 ± 0.29	2.52 ± 0.10	2.44 ± 0.77	2.70 ± 0.11	3.67 ± 0.64	5.15 ± 0.65	5.80 ± 0.18	5.15 ± 0.26	4.58 ± 0.09
**Σ**	**5.50**	**7.34**	**8.64**	**8.74**	**7.88**	**10.19**	**15.10**	**15.73**	**14.95**	**13.56**
**TOTAL Methylxanthines g/kg of CI**	**5.50**	**7.34**	**8.64**	**8.74**	**7.88**	**10.19**	**15.10**	**15.73**	**14.95**	**13.56**
**Spectrophotometric assays**										
TPC (g GAE/ Kg of CBS)	3.96 ± 0.34	4.90 ± 0.32	2.89 ± 1.69	2.24 ± 0.05	2.81 ± 0.26	3.23 ± 0.24	3.22 ± 0.13	2.66 ± 0.11	2.56 ± 0.13	3.13 ± 0.08
TFC (g CE/ Kg of CBS)	0.14 ± 0.02	0.48 ± 0.05	0.33 ± 0.04	0.27 ± 0.06	0.45 ± 0.06	0.17 ± 0.04	0.34 ± 0.02	0.77 ± 0.06	0.22 ± 0.04	0.24 ± 0.05
TTC (g CE/ Kg of CBS)	0.32 ± 0.15	0.59 ± 0.04	0.11 ± 0.01	0.19 ± 0.03	0.26 ± 0.11	0.13 ± 0.02	0.25 ± 0.01	0.13 ± 0.01	0.18 ± 0.01	0.28 ± 0.02
RSA (mmol TE/ Kg of CBS)	8.27 ± 0.68	10.79 ± 1.10	5.91 ± 3.61	5.57 ± 0.58	6.04 ± 0.23	7.34 ± 0.84	6.80 ± 0.35	4.92 ± 0.24	4.88 ± 0.18	5.70 ± 0.79

Results expressed as mean values (n = 4) ± standard deviation. The summation (Σ) of chemical compounds is highlighted in bold.

Table S3. Total amounts of bioactive compounds (n = 10) and spectrophotometric assays results determined in fraction F2 yielded from CBS samples from *Trinitario* and *Criollo* cultivars and from different regions of Venezuela. Data are expressed as mg/kg of CBS.

Compound	Sur del Lago_T	Caucagua_T	Merida_1_T	Cuyagua_T	Ocumare_1_T	Canoabo_C	Merida_2_C	Merida_3_C	Carenero_C	Ocumare_2_C
**Phenolic acids**										
Protocatechuic acid	24.95 ± 1.43	22.03 ± 6.09	59.22 ± 11.70	64.50 ± 14.65	42.75 ± 10.58	39.92 ± 9.66	14.47 ± 2.57	28.63 ± 2.60	14.07 ± 4.18	5.51 ± 0.47
**Σ**	**24.95**	**22.03**	**59.22**	**64.50**	**42.75**	**39.92**	**14.47**	**28.63**	**14.07**	**5.51**
**Catechin-3-O-glycosides**										
Catechin-3-O-glucoside_1	77.67 ± 23.46	84.64 ± 33.01	22.70 ± 1.23	90.02 ± 15.74	32.17 ± 3.92	17.70 ± 7.70	17.85 ± 5.33	17.68 ± 8.45	22.51 ± 7.67	33.79 ± 5.13
Catechin-3-O-glucoside_2	1.95 ± 0.67	1.75 ± 0.77	1.49 ± 0.09	6.96 ± 2.98	8.05 ± 3.51	n.q.	5.61 ± 1.73	2.44 ± 0.14	1.22 ± 0.50	0.90 ± 0.16
**Σ**	**79.62**	**86.39**	**24.19**	**96.98**	**40.22**	**17.70**	**23.46**	**20.12**	**23.73**	**34.69**
**Procyanidins B-type (PCB)**										
PCB_1	n.q.	n.q.	1.50 ± 0.62	16.30 ± 8.17	14.42 ± 1.40	11.18 ± 7.90	4.51 ± 0.26	3.04 ± 0.38	5.79 ± 1.72	3.84 ± 0.81
PCB_2	12.57 ± 5.62	21.27 ± 3.28	25.56 ± 2.47	25.23 ± 1.63	17.02 ± 3.96	11.92 ± 4.41	42.359 ± 3.6282	11.69 ± 0.84	8.73 ± 2.39	1.33 ± 0.18
PCB_3	2.45 ± 0.64	n.q.	4.68 ± 0.25	4.10 ± 1.12	2.07 ± 0.49	2.49 ± 1.01	3.58 ± 0.31	1.78 ± 0.55	1.78 ± 0.72	n.q.
**Σ**	**15.02**	**21.27**	**31.75**	**45.62**	**33.51**	**25.58**	**50.45**	**16.51**	**16.30**	**5.17**
**N-Phenylpropenoyl-L-amino acids**										
N-Coumaroyl-L-aspartate_1	8.62 ± 1.13	2.51 ± 0.74	14.90 ± 3.11	10.48 ± 1.70	7.90 ± 2.17	13.65 ± 2.98	6.50 ± 2.37	14.60 ± 6.48	7.73 ± 2.59	2.40 ± 0.24
N-Caffeoyl-L-aspartate	0.29 ± 0.08	0.82 ± 0.20	7.22 ± 0.94	2.40 ± 0.31	3.84 ± 0.65	2.40 ± 0.63	1.05 ± 0.10	n.q.	n.q.	n.q.
N-Coumaroyl-L-aspartate_2	n.q.	n.q.	3.92 ± 0.47	1.15 ± 0.05	1.12 ± 0.29	1.50 ± 0.43	0.68 ± 0.13	1.22 ± 0.16	3.69 ± 0.91	0.39 ± 0.04
**Σ**	**8.90**	**3.32**	**26.03**	**14.03**	**12.87**	**17.55**	**8.24**	**15.83**	**11.41**	**2.79**
**Other**										
C11H21O9S_1	10.18 ± 1.82	3.36 ± 1.35	10.66 ± 0.39	16.98 ± 9.45	21.23 ± 3.34	8.14 ± 5.76	13.45 ± 3.40	12.23 ± 2.38	9.20 ± 2.79	3.14 ± 0.47
**Σ**	**10.18**	**3.36**	**10.66**	**16.98**	**21.23**	**8.14**	**13.45**	**12.23**	**9.20**	**3.14**
**Σ Total (mg/kg of CBS)**	**138.68**	**136.38**	**151.85**	**238.12**	**150.57**	**108.89**	**110.07**	**93.31**	**74.71**	**51.29**
**Spectrophotometric assays**										
TPC (g GAE/ kg of CBS)	1.03 ± 0.08	1.79 ± 0.08	1.10 ± 0.09	1.21 ± 0.12	1.33 ± 0.06	1.03 ± 0.03	1.38 ± 0.11	1.53 ± 0.08	1.16 ± 0.03	1.34 ± 0.10
TFC (g CE/ kg of CBS)	0.56 ± 0.04	0.98 ± 0.02	0.68 ± 0.07	0.69 ± 0.15	0.75 ± 0.06	0.62 ± 0.05	0.62 ± 0.05	0.77 ± 0.06	0.46 ± 0.02	0.49 ± 0.04
TTC (g CE/ kg of CBS)	0.17 ± 0.07	0.30 ± 0.07	0.13 ± 0.01	0.14 ± 0.01	0.09 ± 0.03	0.07 ± 0.03	0.12 ± 0.01	0.13 ± 0.01	0.10 ± 0.00	0.10 ± 0.02
RSA (mmol TE/ kg of CBS)	3.77 ± 0.29	5.76 ± 0.16	3.73 ± 0.29	4.36 ± 0.44	4.49 ± 0.08	3.93 ± 0.11	4.18 ± 0.33	4.92 ± 0.24	3.70 ± 0.11	4.07 ± 0.43

Results expressed as mean values (n = 4) ± standard deviation. The summation (Σ) of chemical compounds is highlighted in bold.

Table S4. Total amounts of bioactive compounds (n = 23) and spectrophotometric assays results determined in fraction F3 yielded from CBS samples from *Trinitario* and *Criollo* cultivars and from different regions of Venezuela. Data are expressed mg/kg of CBS.

Compound	Sur del Lago_T	Caucagua_T	Merida_1_T	Cuyagua_T	Ocumare_1_T	Canoabo_C	Merida_2_C	Merida_3_C	Carenero_C	Ocumare_2_C
**Phenolic acids**										
Protocatechuic acid	44.68 ± 8.25	31.92 ± 11.81	43.83 ± 6.66	49.34 ± 1.82	41.38 ± 7.50	71.44 ± 18.67	26.65 ± 8.58	18.25 ± 5.31	13.30 ± 2.93	6.56 ± 0.80
**Σ**	**44.68**	**31.92**	**43.83**	**49.34**	**41.38**	**71.44**	**26.65**	**18.25**	**13.30**	**6.56**
**Flavan-3-ols**										
Catechin	17.04 ± 4.72	16.27 ± 3.42	48.31 ± 4.76	46.87 ± 9.88	32.43 ± 1.40	22.05 ± 5.04	10.73 ± 2.37	11.08 ± 2.41	6.04 ± 1.37	5.04 ± 1.09
Epicatechin	170.88 ± 40.19	102.27 ± 29.04	298.00 ± 234.20	234.20 ± 7.87	200.78 ± 13.59	140.67 ± 32.69	65.61 ± 15.58	61.00 ± 13.51	30.59 ± 7.11	31.23 ± 7.40
**Σ**	**187.92**	**118.54**	**346.31**	**281.08**	**233.21**	**162.72**	**76.34**	**72.07**	**36.64**	**36.27**
**Procyanidins B-type (PCB)**										
PCB_1	5.32 ± 1.84	3.57 ± 0.70	2.44 ± 0.32	1.94 ± 0.33	1.85 ± 0.39	3.03 ± 1.27	10.85 ± 6.80	1.10 ± 0.23	0.69 ± 0.07	n.q.
PCB_3	2.61 ± 0.60	2.29 ± 0.58	0.24 ± 0.01	2.01 ± 0.53	1.36 ± 0.29	1.23 ± 0.35	0.89 ± 0.31	0.59 ± 0.17	0.36 ± 0.04	0.56 ± 0.15
PCB_4	5.03 ± 0.38	5.48 ± 2.24	5.40 ± 2.04	8.54 ± 5.53	10.81 ± 2.70	3.97 ± 0.92	2.61 ± 0.99	1.13 ± 0.51	0.85 ± 0.17	1.18 ± 0.20
**Σ**	**12.97**	**11.34**	**8.08**	**12.48**	**14.02**	**8.23**	**14.34**	**2.81**	**1.90**	**1.73**
**Procyanidins B-type (PCB) trimers**										
PCB trimer_5	8.09 ± 2.34	10.46 ± 2.38	3.67 ± 0.54	2.95 ± 0.98	4.81 ± 0.63	4.75 ± 1.25	7.60 ± 3.90	1.85 ± 0.77	1.27 ± 0.22	1.28 ± 0.24
**Σ**	**8.09**	**10.46**	**3.67**	**2.95**	**4.81**	**4.75**	**7.60**	**1.85**	**1.27**	**1.28**
**Procyanidins A-type (PCA) glycosides**										
PCA pentoside_1	1.32 ± 0.51	2.32 ± 0.50	1.89 ± 0.13	2.00 ± 0.38	1.94 ± 0.48	1.69 ± 0.15	0.88 ± 0.12	0.84 ± 0.45	0.42 ± 0.02	0.59 ± 0.18
PCA pentoside_2	4.52 ± 0.57	3.56 ± 1.12	11.65 ± 1.71	8.96 ± 6.85	9.55 ± 5.64	4.09 ± 2.60	3.08 ± 1.74	3.18 ± 0.67	2.03 ± 0.36	1.67 ± 0.54
PCA pentoside_3	4.52 ± 0.57	3.56 ± 1.12	11.65 ± 1.71	6.47 ± 2.26	5.75 ± 1.11	6.47 ± 2.26	5.64 ± 0.89	4.47 ± 0.94	4.05 ± 0.91	1.83 ± 0.71
PCA hexoside_1	n.q.	n.q.	n.q.	n.q.	n.q.	n.q.	22.42 ± 4.00	14.46 ± 3.66	11.43 ± 2.16	1.03 ± 0.05
PCA hexoside_2	9.11 ± 1.95	8.73 ± 4.83	21.70 ± 2.03	15.05 ± 4.42	15.08 ± 2.20	14.17 ± 2.90	5.73 ± 2.11	6.32 ± 1.50	4.63 ± 0.97	1.95 ± 0.64
**Σ**	**19.46**	**18.17**	**46.88**	**32.49**	**32.31**	**26.43**	**37.75**	**29.27**	**22.56**	**7.08**
**Flavonol-3-O-glycosides**										
Quercetin-3-glucoside	5.86 ± 1.16	9.06 ± 2.50	21.92 ± 1.05	16.70 ± 2.27	21.38 ± 2.90	10.88 ± 2.22	4.98 ± 1.03	5.05 ± 1.16	1.45 ± 0.36	1.36 ± 0.34
Quercetin-3-arabinoside	6.57 ± 1.28	9.04 ± 2.52	28.63 ± 1.89	18.63 ± 2.08	24.33 ± 2.61	15.73 ± 2.71	3.99 ± 0.88	5.86 ± 1.22	2.73 ± 0.67	1.57 ± 0.41
Kaempferol-3-rutinoside	n.q.	1.29 ± 0.36	8.31 ± 0.46	n.q.	n.q.	n.q.	4.08 ± 0.89	2.85 ± 0.80	1.64 ± 0.51	0.21 ± 0.07
**Σ**	**12.42**	**19.38**	**58.86**	**35.34**	**45.70**	**26.60**	**13.05**	**13.76**	**5.82**	**3.14**
**N-Phenylpropenoyl-L-amino acids**										
N-Caffeoyl-L-aspartate	0.69 ± 0.11	2.02 ± 0.84	15.26 ± 0.27	8.01 ± 3.64	13.67 ± 2.41	10.16 ± 2.95	2.41 ± 0.72	1.84 ± 0.60	0.24 ± 0.05	0.13 ± 0.01
N-Coumaroyl-L-aspartate_2	0.93 ± 0.32	1.60 ± 0.91	14.28 ± 0.52	7.03 ± 4.01	14.14 ± 1.17	10.81 ± 2.99	0.32 ± 0.07	0.15 ± 0.04	0.15 ± 0.03	0.11 ± 0.01
N-Coumaroyl-L-glutamate	0.63 ± 0.14	0.59 ± 0.12	1.18 ± 0.01	1.25 ± 0.56	1.23 ± 0.18	1.17 ± 0.18	0.72 ± 0.49	0.36 ± 0.09	0.22 ± 0.05	0.13 ± 0.02
N-Feruloyl-L-aspartate	0.74 ± 0.32	1.01 ± 0.35	2.47 ± 0.28	2.16 ± 0.75	2.89 ± 0.40	1.56 ± 0.55	1.06 ± 0.27	0.74 ± 0.14	0.31 ± 0.03	0.14 ± 0.02
N-Coumaroyl-L-tyrosine	1.35 0.41	1.42 0.72	2.38 0.40	1.50 0.39	1.86 0.24	1.79 0.36	1.46 0.49	1.03 0.22	0.70 0.14	0.29 0.06
**Σ**	**4.33**	**6.64**	**35.58**	**19.95**	**33.78**	**25.49**	**5.97**	**4.11**	**1.61**	**0.81**
**Other**										
C11H21O9S_2	9.04 ± 4.94	15.55 ± 4.88	11.62 ± 2.23	20.23 ± 4.38	26.48 ± 2.68	9.27 ± 2.93	13.42 ± 3.18	4.01 ± 1.38	6.40 ± 1.95	1.14 ± 0.46
Hydroxyjasmonic acid sulfate	3.51 ± 0.33	14.42 ± 6.64	2.82 ± 0.76	2.33 ± 0.32	9.05 ± 1.39	23.17 ± 5.43	14.41 ± 7.20	11.63 ± 5.38	2.88 ± 0.98	1.01 ± 0.30
Sweroside	4.89 ± 1.06	6.01 ± 2.91	11.75 ± 1.83	22.12 ± 12.44	15.83 ± 3.72	5.78 ± 1.75	7.18 ± 1.49	2.15 ± 0.39	1.17 ± 0.25	1.23 ± 0.40
**Σ**	**17.44**	**35.98**	**26.20**	**44.68**	**51.37**	**38.22**	**35.01**	**17.79**	**10.46**	**3.39**
**Σ Total Polyphenols (ug/g)**	**307.31**	**252.43**	**569.41**	**478.31**	**456.58**	**363.87**	**216.72**	**159.92**	**93.55**	**60.25**
**Spectrophotometric assays**										
TPC (mg GAE g-1 of CBS)	0.89 ± 0.14	1.07 ± 0.15	1.14 ± 0.04	1.08 ± 0.06	1.10 ± 0.09	0.80 ± 0.16	1.11 ± 0.02	1.28 ± 0.05	0.78 ± 0.07	1.04 ± 0.03
TFC (mg CE g-1 of CBS)	0.65 ± 0.10	0.71 ± 0.09	0.89 ± 0.08	0.80 ± 0.08	0.71 ± 0.08	0.60 ± 0.14	0.66 ± 0.01	0.82 ± 0.03	0.46 ± 0.05	0.59 ± 0.03
TTC (mg CE g-1 of CBS)	0.27 ± 0.04	0.26 ± 0.01	0.47 ± 0.04	0.30 ± 0.08	0.31 ± 0.10	0.22 ± 0.05	0.46 ± 0.05	0.76 ± 0.06	0.40 ± 0.02	0.72 ± 0.05
RSA (umol TE g-1 og CBS)	3.51 ± 0.46	3.87 ± 0.47	4.21 ± 0.20	4.24 ± 0.30	4.06 ± 0.22	3.23 ± 0.55	4.12 ± 0.05	4.86 ± 0.23	3.03 ± 0.21	3.91 ± 0.28

Results expressed as mean values (n = 4) ± standard deviation. The summation (Σ) of chemical compounds is highlighted in bold.

Table S5. Total amounts of bioactive compounds (n=19) and spectrophotometric assays results determined in fraction F4 yielded from CBS samples from *Trinitario* and *Criollo* cultivars and from different regions of Venezuela. Data are expressed as mg/kg of CBS.

Compound	Sur del Lago_T	Caucagua_T	Merida_1_T	Cuyagua_T	Ocumare_1_T	Canoabo_C	Merida_2_C	Merida_3_C	Carenero_C	Ocumare_2_C
**Procyanidins B-type (PCB)**										
PCB_1	31.31 ± 9.84	17.30 ± 4.82	44.53 ± 0.77	27.67 ± 4.45	25.88 ± 2.96	23.58 ± 8.07	19.58 ± 8.53	21.68 ± 5.69	14.82 ± 4.93	2.40 ± 1.09
**Σ**	**31.31**	**17.30**	**44.53**	**27.67**	**25.88**	**23.58**	**19.58**	**21.68**	**14.82**	**2.40**
**Procyanidins B-type (PCB) trimers**										
PCB trimer_1	4.68 ± 1.58	7.22 ± 1.89	9.32 ± 1.37	14.27 ± 1.15	13.95 ± 0.76	6.06 ± 0.79	8.44 ± 2.62	7.05 ± 2.75	4.36 ± 1.77	3.02 ± 1.35
PCB trimer_2	78.93 ± 21.89	45.92 ± 12.72	130.53 ± 15.03	99.60 ± 6.92	79.64 ± 4.88	59.52 ± 11.26	49.22 ± 7.87	40.67 ± 10.30	38.43 ± 13.35	46.11 ± 8.21
PCB trimer_3	4.19 ± 3.12	n.q.	n.q.	n.q.	n.q.	2.18 ± 0.67	16.64 ± 8.75	22.65 ± 8.20	13.98 ± 5.02	5.43 ± 3.62
PCB trimer_4	58.78 ± 14.04	21.07 ± 6.01	78.01 ± 10.12	47.46 ± 6.41	38.17 ± 2.68	39.53 ± 8.58	2.96 ± 4.28	3.30 ± 3.14	0.74 ± 0.79	0.40 ± 0.44
PCB trimer_6	49.33 ± 9.95	18.16 ± 5.37	51.95 ± 11.43	30.70 ± 5.70	22.95 ± 2.43	28.59 ± 6.41	32.50 ± 9.52	33.03 ± 12.50	17.95 ± 2.82	15.81 ± 3.08
PCB trimer_7	8.10 ± 1.68	n.q.	41.51 ± 5.18	18.78 ± 4.43	22.13 ± 1.08	6.42 ± 1.41	2.89 ± 0.22	1.37 ± 0.38	1.37 ± 0.56	0.99 ± 0.40
PCB trimer_8	21.49 ± 3.52	11.91 ± 3.86	31.49 ± 3.99	20.02 ± 3.55	22.33 ± 4.01	17.89 ± 1.72	12.66 ± 5.26	13.60 ± 3.70	10.40 ± 2.54	4.62 ± 0.56
**Σ**	**225.51**	**104.27**	**342.81**	**230.83**	**199.18**	**160.20**	**125.31**	**121.68**	**87.22**	**76.38**
**Procyanidins A-type (PCA) glycosides**										
PCA pentoside_2	8.18 ± 1.62	8.11 ± 2.12	9.02 ± 3.55	9.51 ± 1.22	11.53 ± 1.51	9.12 ± 1.59	15.43 ± 5.51	25.36 ± 12.38	14.71 ± 5.18	6.97 ± 2.38
PCA pentoside_3	10.22 ± 1.41	4.87 ± 2.40	27.42 ± 1.48	12.47 ± 1.97	12.21 ± 2.27	17.24 ± 2.22	47.13 ± 14.23	67.13 ± 13.83	43.27 ± 7.57	13.20 ± 3.65
PCA hexoside_2	9.75 ± 1.87	2.97 ± 0.75	15.41 ± 2.28	6.30 ± 1.04	9.68 ± 1.23	8.81 ± 1.66	7.43 ± 0.74	3.29 ± 0.44	8.68 ± 2.00	1.813 ± 0.703
PCA trimer arabinoside	24.03 ± 1.65	12.85 ± 3.40	24.56 ± 2.53	17.73 ± 3.08	16.76 ± 2.38	15.38 ± 2.94	15.29 ± 3.63	16.91 ± 5.76	12.09 ± 5.86	5.78 ± 1.17
**Σ**	**52.18**	**28.80**	**76.41**	**46.02**	**50.19**	**50.55**	**85.28**	**112.70**	**78.76**	**27.76**
**Flavonol-3-O-glycosides**										
Quercetin-3-arabinoside	5.05 ± 1.29	5.83 ± 1.84	16.83 ± 0.87	9.19 ± 1.27	11.33 ± 1.45	10.77 ± 1.13	3.34 ± 1.51	8.62 ± 1.72	3.81 ± 0.88	1.74 ± 0.52
**Σ**	**5.05**	**5.83**	**16.83**	**9.19**	**11.33**	**10.77**	**3.34**	**8.62**	**3.81**	**1.74**
**Flavonols**										
Quercetin	1.76 ± 0.61	1.94 ± 0.34	2.88 ± 0.49	2.59 ± 0.18	4.51 ± 0.49	1.92 ± 0.34	0.91 ± 0.07	0.98 ± 0.03	0.83 ± 0.02	0.75 ± 0.01
**Σ**	**1.76**	**1.94**	**2.88**	**2.59**	**4.51**	**1.92**	**0.91**	**0.98**	**0.83**	**0.75**
**N-Phenylpropenoyl-L-amino Acids**										
N-Caffeoyl-L-aspartate	4.35 ± 3.21	13.38 ± 3.71	74.77 ± 6.13	93.86 ± 4.46	93.96 ± 2.44	13.38 ± 5.11	66.11 ± 19.01	67.84 ± 3.15	28.11 ± 2.08	15.33 ± 4.22
N-Coumaroyl-L-aspartate_2	6.70 ± 3.25	12.01 ± 3.27	46.03 ± 5.12	57.72 ± 5.78	64.05 ± 1.46	15.80 ± 3.43	0.12 ± 0.09	0.08 ± 0.03	0.08 ± 0.03	0.08 ± 0.02
N-Feruloyl-L-aspartate	1.19 ± 0.41	1.44 ± 0.36	3.24 ± 0.45	3.72 ± 0.13	3.37 ± 0.25	2.01 ± 0.36	1.09 ± 0.17	2.07 ± 0.58	1.64 ± 0.74	0.77 ± 0.36
N-Coumaroyl-L-tyrosine	8.10 ± 1.03	4.90 ± 1.36	20.73 ± 0.85	7.49 ± 0.42	9.85 ± 0.35	16.01 ± 0.86	12.19 ± 4.96	7.94 ± 1.53	6.02 ± 0.49	6.80 ± 0.19
**Σ**	**20.35**	**31.74**	**144.77**	**162.78**	**171.23**	**47.21**	**79.50**	**77.94**	**35.85**	**22.97**
**Other**										
C11H21O9S_2	4.86 ± 1.87	8.13 ± 2.60	6.88 ± 1.90	13.31 ± 1.11	15.34 ± 1.56	5.36 ± 3.04	30.26 ± 19.50	8.54 ± 3.55	15.94 ± 8.01	5.40 ± 2.59
**Σ**	**4.86**	**8.13**	**6.88**	**13.31**	**15.34**	**5.36**	**30.26**	**8.54**	**15.94**	**5.40**
**Σ Total (mg/kg of CBS)**	**341.03**	**198.03**	**635.11**	**492.39**	**477.66**	**299.58**	**344.18**	**352.14**	**237.23**	**137.41**
**Spectrophotometric assays**										
TPC (g GAE/ kg of CBS)	1.64 ± 0.20	1.37 ± 0.17	2.28 ± 0.13	1.93 ± 0.18	2.10 ± 0.12	1.30 ± 0.10	1.68 ± 0.26	2.34 ± 0.32	1.37 ± 0.19	2.21 ± 0.34
TFC (g CE/ kg of CBS)	1.19 ± 0.16	0.97 ± 0.15	1.75 ± 0.09	1.55 ± 0.20	1.38 ± 0.11	0.80 ± 0.23	0.95 ± 0.14	1.45 ± 0.22	0.74 ± 0.11	1.18 ± 0.19
TTC (g CE/ kg of CBS)	0.65 ± 0.19	0.44 ± 0.12	1.05 ± 0.09	0.67 ± 0.14	0.65 ± 0.17	0.52 ± 0.07	0.23 ± 0.01	0.11 ± 0.01	0.17 ± 0.00	0.12 ± 0.04
RSA (mmol TE/ kg of CBS)	7.67 ± 0.81	5.91 ± 0.69	9.80 ± 0.58	9.18 ± 0.96	8.98 ± 0.15	7.19 ± 3.37	6.70 ± 0.91	9.40 ± 0.92	5.71 ± 0.58	9.23 ± 1.22

Results expressed as mean values (n = 4) ± standard deviation. The summation (Σ) of chemical compounds is highlighted in bold.
